# Rising tides: Unveiling the spatial and temporal evolution of sea level rise under climate change

**DOI:** 10.1371/journal.pone.0347855

**Published:** 2026-06-17

**Authors:** Bing Liang, Guoqing Shi, Yuxuan Zhu, Yuexi Wu, Mark Wang

**Affiliations:** 1 Business School, Wuxi Taihu University, Wuxi, China; 2 Centre for Contemporary Chinese Studies, University of Melbourne, Parkville, Australia; 3 National Research Center for Resettlement, Hohai University, Nanjing, China; 4 College of Geography and Remote Sensing, Hohai University, Nanjing, China; Universidade de Aveiro, PORTUGAL

## Abstract

This study systematically investigates the spatiotemporal evolution of sea level rise under climate change, employing a tri-scale quantitative framework (global, China’s coastal waters, and Shanghai municipality) to elucidate its underlying drivers and regional disparities. By synthesizing IPCC AR6 datasets and NASA sea level projection models, we integrate the Theil-Sen Median Method with Mann-Kendall Test to analyze trajectory patterns from 2030 to 2100. Spatial clustering effects are further identified through hotspot analysis (Getis-Ord Gi*) implemented in ArcGIS Pro. The findings reveal a statistically significant upward trend in global sea levels, primarily attributed to thermal expansion and cryospheric melt (glaciers and polar ice sheets), with localized subsidence observed in certain high-latitude regions. China’s coastal waters exhibit accelerated sea level rise, particularly in the South China Sea and East China Sea, where rates surpass the global mean—a phenomenon driven by coupled effects of monsoon circulation, Kuroshio Current dynamics, and freshwater discharge from major rivers (e.g., Yangtze and Yellow Rivers). At the urban scale, Shanghai’s coastal zone demonstrates exacerbated relative sea level rise due to superimposed land subsidence and localized hydrodynamic processes, manifesting distinct spatiotemporal clustering patterns. By integrating global-scale thermodynamic baselines, regional oceanic drivers, and local land subsidence patterns, this study provides a quantitative foundation for place-based adaptation strategies in delta cities. The findings enable evidence-based risk assessment and inform anticipatory governance measures—such as targeted infrastructure reinforcement and land-use planning adjustments—to address the compound sea level risks identified at each scale.

## 1. Introduction

Climate change stands as one of the most critical challenges confronting humanity in the 21st century, with cascading impacts permeating natural environments, socioeconomic systems, and global ecological networks [1].Among these impacts, Sea Level Rise (SLR)—a direct consequence of climate change—has emerged as a focal point of global concern [2].The Intergovernmental Panel on Climate Change (IPCC) Sixth Assessment Report (AR6) substantiates that global mean sea level has risen approximately 20 centimeters since 1900, with the rate of increase accelerating from 1.4 mm/year in the early 20th century to 3.6 mm/year in the early 21st century [3]. Under high-emission scenarios, projections indicate a potential rise of 0.63 to 1.01 meters by 2100, posing unprecedented risks to coastal zones worldwide [4]. SLR not only jeopardizes coastal ecosystems and human settlements but also amplifies its destructive impacts through intensified extreme weather events and accelerated coastal erosion [5,6]. Consequently, deciphering the spatiotemporal dynamics and underlying drivers of SLR has become an imperative for developing effective climate change mitigation and adaptation strategies.

**Fig 1:**
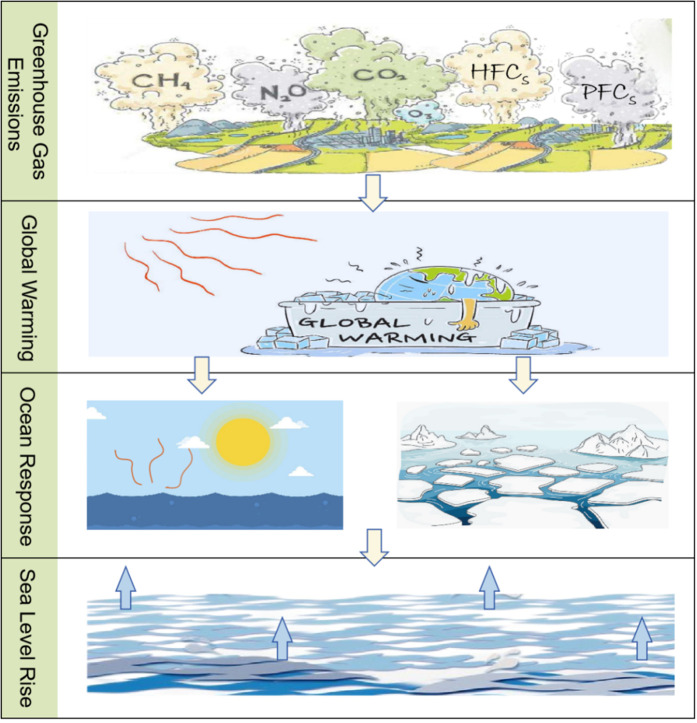
Schematic Diagram of SLR.

The primary drivers of SLR stem from two climate-induced physical processes: thermal expansion of seawater and land ice melt [7]. Thermal expansion occurs as global warming increases ocean temperatures and volume, whereas land ice melt predominantly originates from the ablation of Greenland and Antarctic ice sheets coupled with mountain glacier retreat [8]. Regional compounding factors—including land subsidence, groundwater extraction, and ocean dynamic processes (e.g., current variations)—further amplify SLR magnitudes at local scales [9]. The interactive effects of these drivers result in marked spatiotemporal heterogeneity, manifesting as divergent rates and impact intensities across geographical regions and temporal scales [10]. For instance, Asian deltaic regions and Pacific Small Island Developing States (SIDS), characterized by low-lying topography and enhanced subsidence, experience disproportionately higher SLR impacts compared to global averages [11]. See Fig 1 for detailed mechanistic pathways.

SLR generates multidimensional consequences that directly disrupt ecological, social, and economic systems in coastal zones [12]. Primarily, SLR induces permanent inundation of low-lying areas and progressive coastline erosion, jeopardizing critical infrastructure and human security in coastal communities [13]. Projections indicate that hundreds of millions worldwide will face heightened flood risks by 2100, with disproportionate exposure in densely populated Asian megacities and climate-vulnerable SIDS. Furthermore, SLR amplifies the destructive potential of storm surges and extreme weather events. For instance, tropical cyclones (e.g., hurricanes and typhoons) under elevated sea levels trigger more extensive inland flooding and compound economic losses [14]. Moreover, saltwater intrusion leads to soil salinization and freshwater resource depletion, imposing chronic threats to agricultural productivity and ecosystem resilience [15]. The synergistic interactions of these cascading impacts position SLR not only as a scientific priority but also as an urgent governance challenge demanding coordinated global policy interventions and localized adaptation strategies.

Under this context, elucidating the Spatiotemporal Evolution characteristics of SLR carries significant scientific and practical implications. The term “Spatiotemporal Evolution” refers to the dynamic variation process of SLR across temporal sequences and spatial distributions [16]. This conceptual framework emphasizes that SLR is not a globally uniform phenomenon, but rather a complex process shaped by the interplay of regional climate patterns, ocean dynamics, and terrestrial surface processes [17]. Comprehending the Spatiotemporal Evolution of SLR enables us to not only decipher its historical variation patterns and future trajectories, but also provides crucial foundations for developing targeted adaptation and mitigation strategies [18]. Practical applications include: identifying SLR high-risk zones to prioritize flood defense infrastructure deployment and population relocation planning; analyzing its temporal evolution patterns to forecast both near-term and long-term risks, thereby informing critical infrastructure design and land-use planning decisions [19].

However, a critical gap persists in the comprehensive understanding of SLR’s spatiotemporal evolution, particularly regarding its high-resolution characterization at local scales. A rich body of literature has extensively documented the impacts of SLR on coastal systems, such as salinity intrusion, inundation extent, and biogeochemical cycles in estuaries and coastal seas worldwide [ 20,21]. These studies are crucial for assessing vulnerability and informing adaptation. Nevertheless, the vast majority of this research treats SLR as a boundary condition—often derived from coarse global or regional models—rather than focusing on the SLR signal itself as a dynamic, spatially heterogeneous process. Consequently, there is a distinct lack of high-resolution analysis that disentangles the localized spatiotemporal evolution of SLR in critical areas like megacities or estuarine zones [22]. For instance, in coastal megacities such as Shanghai, where land subsidence and complex estuarine hydrodynamics interact to amplify local SLR characteristics well beyond global averages, detailed investigations into the precise spatiotemporal patterns of this amplified rise remain insufficient [23].

Building upon the aforementioned context, this study aims to systematically unravel the spatiotemporal evolution characteristics of SLR under climate change through multi-source data fusion and advanced numerical modeling. Specifically, the research objectives include: (1) Quantifying regional disparities and temporal evolution patterns of SLR using spatiotemporal analytics; (2) Assessing SLR impacts and proposing adaptive governance frameworks through case studies of representative coastal cities (e.g., Shanghai).The innovation of this study lies in integrating multi-source datasets with high-resolution modeling to achieve the first systemic characterization of SLR’s spatiotemporal evolution, while pioneering urban-scale applications to inform localized climate adaptation. As a critical manifestation of climate change, deciphering SLR’s spatiotemporal evolution is essential for understanding its drivers, projecting future trajectories, and formulating mitigation strategies.

## 2. Methods

### 2.1. Study area

**Fig 2:**
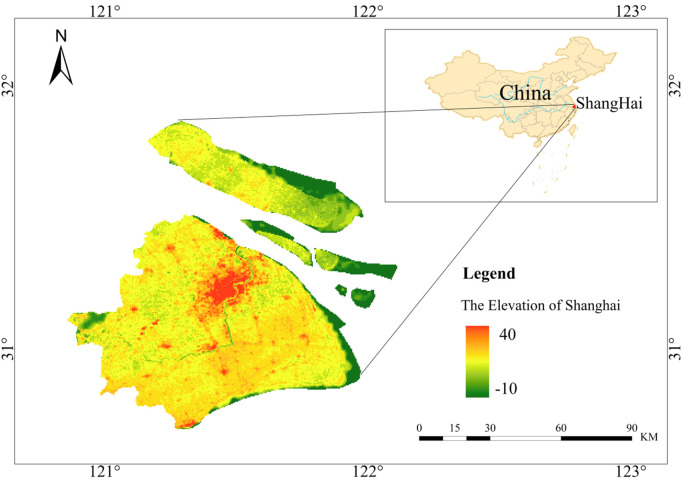
Overview Map of Shanghai. Maps from the Ministry of Natural Resources of the People’s Republic of China, approval number is GS (2016) 2923.

Shanghai is situated on the eastern coast of China at the southern mouth of the Yangtze River, forming the core of the Yangtze River Delta (YRD) alluvial plain (Fig 2) [24]. Its geographical location makes it one of the most prominent megacities globally that is acutely vulnerable to climate-induced SLR.

The city’s topography is characteristically low and flat, with an average elevation of approximately 4 meters above the mean sea level, and large portions of the urban area lying less than 3.5 meters above sea level. This low-lying topography is a product of its deltaic origins, composed of unconsolidated Holocene sediments that are naturally prone to compaction.

Shanghai experiences a northern subtropical monsoon climate, characterized by distinct seasons and abundant rainfall, with an annual precipitation averaging 1,200 mm. Critically, the local hydrodynamic regime is dominated by a regular semi-diurnal tide. The mean tidal range at the mouth of the Yangtze River is approximately 2.4 to 2.8 meters, with a maximum spring tidal range that can exceed 4.5 meters. This significant tidal amplitude, combined with the funnel-shaped estuary, amplifies storm surges and facilitates the upstream propagation of tidal waves, directly influencing water levels in the Huangpu River that flows through the city center.

The Yangtze River, the longest river in Asia, discharges an enormous volume of freshwater and sediment into the East China Sea via Shanghai. Its multi-annual mean freshwater discharge is approximately 900 billion cubic meters per year, with a sediment load that historically exceeded 400 million tons annually. This massive freshwater inflow creates a distinct salinity gradient in the coastal waters, influencing density-driven circulation and, consequently, local steric sea level patterns. However, the construction of major upstream dams, most notably the Three Gorges Dam, has drastically reduced the sediment supply to the delta by over 70% in recent decades. This sediment starvation exacerbates the relative SLR by inhibiting natural land accretion and potentially accelerating subsidence.

Furthermore, Shanghai is afflicted by significant land subsidence, a critical compounding factor for relative SLR. Driven by a century of extensive groundwater extraction for industrial and domestic use, as well as the immense load of its urban infrastructure, some areas of the city have experienced cumulative subsidence exceeding 3 meters since the 1920s. Although extraction rates have been regulated, residual compaction and ongoing urban construction continue to cause a subsidence rate of several millimeters per year in many districts.

As a global financial and transportation hub with over 24 million residents, Shanghai’s low elevation, dense population, and high-value infrastructure make it a global priority case for understanding and adapting to the multiscale drivers of SLR. The city’s vulnerability is not merely a function of global climate change, but is profoundly shaped by the dynamic interplay of its local tidal regime, riverine inputs, and anthropogenic subsidence.

### 2.2. Data sources

This study synthesizes multi-source datasets to analyze the spatiotemporal evolution of SLR across global, regional, and urban scales. The primary data sources and their characteristics are as follows:

① IPCC AR6 Sea Level Projection Dataset

The global and regional sea level projections are derived from the Sixth Assessment Report (AR6) of the Intergovernmental Panel on Climate Change (IPCC), specifically from the datasets underpinning Working Group I’s Chapter 9 (“Ocean, Cryosphere, and Sea Level Change”) and the Summary for Policymakers. The IPCC AR6 provides sea level projections for five Shared Socioeconomic Pathways (SSPs): SSP1–1.9, SSP1–2.6, SSP2–4.5, SSP3–7.0, and SSP5–8.5. These scenarios depict various development pathways of future economic and social systems, reflecting the correlation between societal development and the challenges of mitigating and adapting to climate change. They serve as the core foundation for conducting climate change impact assessments and climate policy formulation. In this study, we primarily use sea level projection data under the intermediate emission scenario SSP2–4.5 for analysis.

Spatial Coverage: Global ocean, with regional breakdowns including China’s coastal seas.

Spatial Resolution: Data are provided on a 1° × 1° latitude-longitude grid for global fields; regional mean time series are also available for predefined coastal segments.

Temporal Coverage: 2020–2100 (with extensions to 2150 for some scenarios).

Temporal Resolution: Annual mean sea level anomalies relative to a 1995–2014 baseline.

Data Components: The dataset includes total SLR as well as contributions from its primary components: thermal expansion, glacier mass loss, Greenland ice sheet melt, Antarctic ice sheet melt, and land water storage changes.

② NASA Sea Level Projection Tool

To complement the IPCC AR6 data and facilitate site-specific analysis, we also utilized the NASA Sea Level Projection Tool, which visualizes and distributes the AR6 scenario outputs alongside observational tide gauge records. The data were accessed via the NASA Sea Level Change Portal.

Data Source: The tool integrates IPCC AR6 projections with NASA’s observational datasets for validation. The underlying model outputs are consistent with the IPCC AR6 data described above.

Query Parameters: For the Shanghai urban-scale analysis, we extracted projections for the grid cell corresponding to the Yangtze River Estuary (approximately 31.5°N, 122.0°E).

Spatial Resolution: The tool provides data at the native 1° × 1° grid resolution of the underlying models, with the ability to extract time series for specific coordinates.

Temporal Coverage and Resolution: Same as the IPCC AR6 dataset (2020–2100, annual means).

Access: The data were retrieved from: https://sealevel.nasa.gov/ipcc-ar6-sea-level-projection-tool.

It is important to clarify that the IPCC AR6 and NASA sea level projection datasets used in this study provide projections of absolute sea level rise—the change in ocean surface height relative to the geocentric reference frame. These global and regional climate models do not, by design, incorporate localized anthropogenic processes such as groundwater extraction-induced subsidence, sediment compaction from urban loading, or other human-driven land surface changes.

### 2.3. Research methodology

#### 2.3.1. SLR temporal evolution trend analysis method.

To robustly quantify the temporal trends in SLR across different scales while accounting for potential non-linearities and data variability, we employed a combination of the Theil-Sen Median Estimator and the Mann-Kendall (MK) test. This non-parametric approach is particularly well-suited for climate time series analysis as it makes no assumptions about the underlying data distribution and is resistant to the influence of outliers and short-term fluctuations.

(1) Theil-Sen Median Estimator for Trend Magnitude

The magnitude of the SLR trend over the 2030–2100 period was estimated using the Theil-Sen Median Estimator (also known as Sen’s slope). This method computes the slope between all possible pairs of data points in the time series and takes the median of these pairwise slopes as a robust estimator of the overall trend. For a time series consisting of data points (xi,yi) and (xj,yj), where x represents the year and y denotes the projected sea level height, the slope βij between any two points with i < j is calculated as:


$$\begin{array}{c} \begin{array}{c} \beta \text{ij}=\frac{y_{j}-y_{i}}{x_{j}-x_{i}}\; \end{array} \end{array}$$ (1-1)

Rank all pairwise-computed slopes and identify the median value, which is defined as Sen’s slope (β_Sen_). The Sen’s slope estimator is formulated as:


$$\begin{array}{c} \begin{array}{c} \beta_{Sen}=\text{Median}(\beta_{ij}) \end{array} \end{array}$$ (1-2)

A positive β_Sen_ indicates an increasing trend over time, while a negative value suggests a decreasing trend. The median-based approach confers significant robustness: unlike ordinary least squares regression—which can be disproportionately influenced by a small number of anomalous years—the Theil-Sen estimator effectively mitigates the impact of outliers and short-term variability, providing a reliable measure of the underlying long-term trend magnitude.

While SLR exhibits a clear long-term increasing trend under climate change, the annual mean sea level time series used in this study (2030–2100) are not perfectly smooth monotonic functions. They contain inherent variability arising from multiple sources. First, interannual to decadal climate modes such as El Niño-Southern Oscillation (ENSO) and Pacific Decadal Oscillation (PDO) can introduce transient fluctuations in regional and even global mean sea level, effectively acting as “noise” superimposed on the underlying trend. Second, in regional-scale analyses (e.g., China’s coastal seas), local processes such as monsoon intensity variations, freshwater discharge anomalies, and ocean current meandering can cause short-term departures from the long-term trajectory. Third, although we utilize multi-model ensemble means from IPCC AR6 to reduce individual model biases, the ensemble averaging process does not completely eliminate all forms of variability, and some grid cells may exhibit values that deviate from the surrounding regional pattern due to unresolved model physics or internal variability.

Given these characteristics, the Theil-Sen Median Estimator is particularly well-suited for this analysis. Unlike ordinary least squares regression, which is highly sensitive to outliers and can be disproportionately influenced by a small number of anomalous years, Theil-Sen computes the median of all pairwise slopes. This approach effectively mitigates the influence of short-term fluctuations and potential data anomalies, providing a robust estimate of the underlying long-term trend. The subsequent Mann-Kendall test, also non-parametric and distribution-free, complements this approach by assessing the statistical significance of the detected trend without assuming normality of the residuals. Together, these methods ensure that our identified trend categories (from “Highly Significant Increase” to “Highly Significant Decrease”) reflect genuine long-term changes rather than being artifacts of interannual variability or data noise.

(2) Mann-Kendall Test for Trend Significance

To assess the statistical significance of the trends identified by the Theil-Sen estimator, we employed the Mann-Kendall (MK) test, a non-parametric method widely used in environmental and climate sciences. The MK test evaluates whether a time series exhibits a monotonic trend by testing the null hypothesis H_0_ that the data are independent and randomly ordered (i.e., no trend) against the alternative hypothesis H_1_ that a monotonic trend exists.

The test is computed using the MK statistic S, which sums the signs of the differences between all pairs of observations. For a time series X_1_,X_2..._X_n_, the statistic S is defined as:


$$\begin{array}{c} \text{S}=\overset{n}{\underset{j=2}{\sum }}\overset{j-1}{\underset{i=1}{\sum }}\text{sgn}(Xj-Xi) \end{array}$$ (1-3)

where sgn denotes the sign function, defined as:


$$\begin{array}{c} \begin{array}{c} @l\text{sgn}\left({\text{x}}_{\text{j}}-{\text{x}}_{\text{i}}\right)=\left\{ \begin{array}{c} @l+\text{1},\;{\text{x}}_{\text{j}}-{\text{x}}_{\text{i}}>\text{0}\\ \text{0},\;{\text{x}}_{\text{j}}-{\text{x}}_{\text{i}}=\text{0}\\ -\text{1},\;{\text{x}}_{\text{j}}-{\text{x}}_{\text{i}}<\text{0} \end{array}\;\right.\; \end{array} \end{array}$$ (1-4)

Perform Trend Test Using the Standardized Statistic Z. The standardized Z-value is calculated as follows:


$$\begin{array}{c} \begin{array}{c} \text{Z}=\left\{ \begin{array}{c} \frac{\text{S}}{\sqrt{\text{Var}(\text{S})}}\;(\text{S}>\text{0})\\ \text{0}\;(\text{S}=\text{0})\\ \frac{\text{S}+\text{1}}{\sqrt{\text{Var}(\text{S})}}\;(\text{S}<\text{0}) \end{array}\right.\; \end{array} \end{array}$$ (1-5)

where the variance Var(S) is computed as:


$$\begin{array}{c} \begin{array}{c} \text{Var}(\text{S})=\frac{\text{n}(\text{n}-\text{1})(\text{2n}+\text{5})}{\text{18}} \end{array} \end{array}$$ (1-6)

In the formula, n represents the number of data points in the time series.

Based on the combined analysis of Sen’s slope and the MK Test statistic Z, the criteria for trend significance are defined as Table 1:

**Table 1:** Summary of Sen’s Slope and MK Test Results.

β	Z	Trend Category	Trend Characteristics
β > 0	2.58 < Z	4	Highly Significant Increase
	1.96 < Z ≤ 2.58	3	Significant Increase
	1.65 < Z ≤ 1.96	2	Marginally Significant Increase
	Z ≤ 1.65	1	Non-Significant Increase
β = 0	Z	0	No Trend
β < 0	Z ≤ 1.65	−1	Non-Significant Decrease
	1.65 < Z ≤ 1.96	−2	Marginally Significant Decrease
	1.96 < Z ≤ 2.58	−3	Significant Decrease
	2.58 < Z	−4	Highly Significant Decrease

The Sen’s slope calculates the median of pairwise slopes. A positive value indicates an increasing trend over time, while a negative value suggests a decreasing trend. Subsequent computation of the variance and MK Test statistic Z determines the statistical significance of the Sen’s slope. By integrating both methods, we assessed the significance of SLR and analyzed the temporal patterns of SLR in Shanghai.

The analysis utilized projected data from 2030 to 2100. Computations for Sen’s slope and the MK Test were conducted on the MATLAB platform, followed by result visualization in ArcGIS Pro.

It is important to note that the nine trend categories defined in Table 1 represent the full spectrum of possible outcomes from the combined Sen’s slope and MK Test analysis. However, depending on the study area and scale, not all categories may appear in the resulting maps. The presence or absence of specific categories in itself provides valuable information about the regional characteristics of SLR. For instance, the absence of declining trends (categories −1 to −4) in a given area indicates that all locations are experiencing either stable or rising sea levels, with no statistically significant decreases. Conversely, the presence of both increasing and decreasing trends within the same region would suggest complex, heterogeneous dynamics.

#### 2.3.2. Analytical framework for spatiotemporal evolution of SLR.

Spatial heterogeneity analysis was conducted using the Getis-Ord Gi* statistic on the ArcGIS Pro platform. To accommodate the raster-to-vector conversion required for subsequent calculations, the following geoprocessing steps were implemented: Fishnet Creation, Value Extraction to Points,Spatial Join.

The Getis-Ord Gi* statistic is a spatial statistical method designed to detect clusters of high or low values (i.e., hotspots or coldspots) within geospatial datasets. Developed by Getis and Ord (1992), this method identifies statistically significant anomalous zones in spatial data by quantifying deviations between observed values and their neighborhood averages. A region is identified as a hotspot or coldspot if its value—combined with those of its neighboring areas—is significantly higher or lower than the study area’s global mean, respectively.

Computational Steps for the Gi* Statistic, Define Spatial Weight Matrix.The quantification of neighborhood relationships between spatial units can be achieved through distance thresholds, K-nearest neighbors (KNN), contiguity-based adjacency, or other spatial criteria. In this study, the K-nearest neighbors method was selected, with a fixed neighborhood size of 40 adjacent features.

Compute Local Gi* Statistic, For each spatial unit (i,j), the local G_ij_* statistic is calculated as:


$$\begin{array}{c} \begin{array}{c} {\text{ G}}_{\text{ij}}=\frac{\sum_{\text{j}=\text{1}}^{\text{k}}{\text{w}}_{\text{ij}}{\text{z}}_{\text{j}}}{\sum_{\text{j}=\text{1}}^{\text{k}}{\text{w}}_{\text{ij}}} \end{array} \end{array}$$ (1-7)

Where w_ij_ denotes the spatial weight between spatial units i and j, zj represents the standardized observed value at location j.

The Gi statistic is computed as:


$$\begin{array}{c} \begin{array}{c} G_{i*}=\frac{G_{i}-E\left(G_{i}\right)}{SD\left(G_{i}\right)} \end{array} \end{array}$$ (1-8)

Where E(Gi) is the expected value of Gi, SD(Gi) is the standard deviation of Gi.

By setting a significance level (e.g., α = 0.05), the computed Gi value is compared against the critical values of the standard normal distribution to determine whether to reject the null hypothesis H0(i.e., no spatial clustering).

## 3. Results

### 3.1. Temporal evolution of SLR

Table 2 summarizes the Sen’s slope (β) and MK Z-statistic values for key regions across the three scales of analysis. These numerical values confirm that the “Highly Significant Increase” category (red zones) corresponds to β values exceeding approximately 4.5 mm/yr with Z > 6.0, while the declining trends observed in high-latitude regions exhibit β values of −0.8 to −1.2 mm/yr with Z ranging from −1.92 to −2.34, consistent with the “Marginally Significant” to “Significant Decrease” categories. The table also reveals that the highest SLR rates within China’s coastal waters occur in the South China Sea and East China Sea (β ≈ 5.4–5.6 mm/yr), with Shanghai’s coastal grid cell exhibiting a rate of 5.8 mm/yr—approximately 20% higher than the global tropical average.

**Table 2:** Summary of SLR Trends for Key Region.

Region	Sen’s Slope β (mm/yr)	MK Z-Statistic	Trend Category
Global Scale			
Atlantic Ocean(Tropical)	4.8	6.32	Highly Significant Increase
Indian Ocean (Tropical)	5.1	6.45	Highly Significant Increase
Pacific Ocean (Tropical)	4.9	6.28	Highly Significant Increase
Arctic Ocean	−0.8	−1.92	Marginally Significant Decrease
Antarctic Coastal	−1.2	−2.34	Significant Decrease
China’s Coastal Seas			
South China Sea	5.6	6.51	Highly Significant Increase
East China Sea	5.4	6.43	Highly Significant Increase
Bohai Sea	5.0	6.12	Highly Significant Increase
Yellow Sea	5.2	6.28	Highly Significant Increase
Shanghai Coastal Waters			
Yangtze Estuary (grid cell)	5.8	6.58	Highly Significant Increase
Hangzhou Bay	5.7	6.52	Highly Significant Increase

*Note: Trends are calculated for the period 2030–2100 under the SSP2–4.5 scenario.

#### 3.1.1. Global-scale analysis of results.

**Fig 3:**
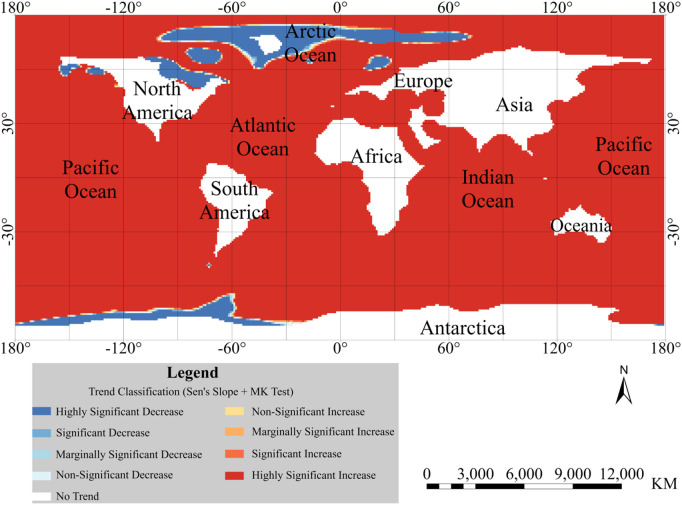
Global SLR Trend (2030–2100).

As illustrated in Fig 3, the projected global SLR trends from 2030 to 2100 are derived from the integration of Sen’s slope estimator, Mann-Kendall Test, and IPCC AR6 emission scenarios.

(figure captions: Maps from the Ministry of Natural Resources of the People’s Republic of China, approval number is GS (2016) 2948)

As shown in Fig 3 above, these visualizations quantify regional variations in SLR trends and their statistical significance levels. The integration of Sen’s slope and the MK Test categorizes the trends into nine distinct classes, as indicated by the legend in the lower-left corner of the figure. It should be noted that not all nine classes are present in the global map; for example, the dark blue category representing “Highly Significant Decrease” is absent, indicating that no oceanic region exhibits a statistically significant declining trend at the global scale. Conversely, the red category (“Highly Significant Increase”) dominates the map, covering over 85% of the global ocean surface. This visual representation reinforces the predominance of accelerating SLR worldwide, while the absence of certain classes in itself conveys meaningful information about the global pattern.

Red zones in the map indicate “Highly Significant Increase”, and the spatial coverage reveals that over 85% of the global ocean exhibits such statistically extreme SLR. These highly significant increase trends are predominantly concentrated across the Atlantic, Indian, and Pacific Oceans, which collectively account for three-quarters of global oceanic surface area. The primary driver of this phenomenon is glacial melting accelerated by global warming, particularly from the Greenland and Antarctic ice sheets [25]. Meltwater influx contributes directly to steric SLR by increasing ocean volume. Additionally, thermal expansion of seawater—resulting from rising ocean temperatures—further elevates sea levels, as heat absorption causes upper-ocean layers to occupy more space [26]. These mechanisms collectively dominate SLR patterns in the Atlantic, Indian, and Pacific basins [27]. Moreover, regional ocean-current dynamics (e.g., the North Atlantic Current) and climate systems (e.g., the Indian Ocean Monsoon System) amplify local SLR rates through feedbacks such as intensified downwelling or altered wind stress [28]. Nevertheless, the global-scale thermodynamic forcing ensures that long-term rising trends remain consistent across all major ocean basins [29].

Blue zones in the map denote “Highly Significant Decrease”, predominantly clustered in high-latitude regions such as the Arctic Ocean, coastal northern North America, and Antarctic marginal seas. These anomalous trends are primarily due to unique cryosphere-hydrosphere interactions under polar amplification of global warming [30]. Specifically: Glacial Isostatic Adjustment (GIA): Near marine-terminating glaciers (e.g., Jakobshavn Isbræ in Greenland), ice sheet mass loss through calving generates immediate gravitational unloading [31]. This triggers crustal uplift and seafloor rebound, temporarily lowering local sea levels through geophysical feedbacks [32]. Oceanographic Forcing: Changes in Atlantic Meridional Overturning Circulation (AMOC) and polar easterlies alter seawater density distributions [33]. For instance, AMOC slowdown reduces northward heat transport, enhancing surface cooling and contraction in subpolar North Atlantic waters. Halosteric Effects: Freshwater influx from melting ice caps lowers seawater salinity, increasing buoyancy and suppressing steric expansion—a process particularly pronounced in the Beaufort Gyre region [34]. Collectively, these mechanisms dominate the observed localized sea level decline, though such trends remain transient against the backdrop of global mean SLR acceleration.

#### 3.1.2 China-scale analysis of results.

**Fig 4:**
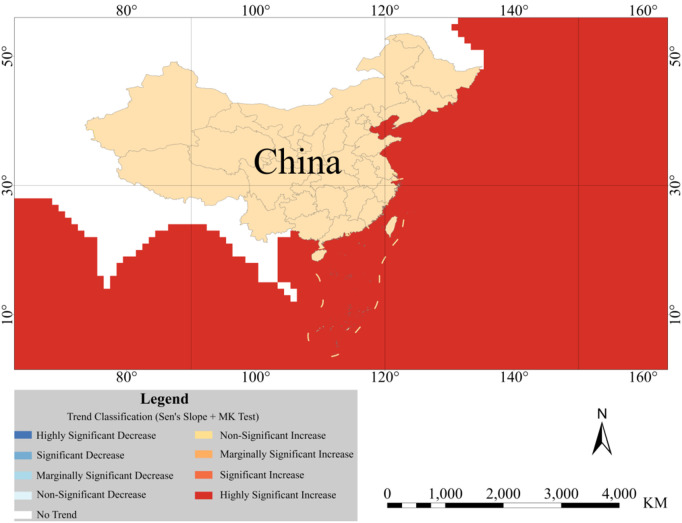
China SLR Trend (2030–2100). Maps from the Ministry of Natural Resources of the People’s Republic of China, approval number is GS (2016) 1600.

As illustrated in Fig 4, the projected China SLR trends from 2030 to 2100 are derived from the integration of Sen’s slope estimator, Mann-Kendall Test, and IPCC AR6 emission scenarios. As with the global analysis, the nine-class trend classification is applied to China’s coastal seas. Notably, the map shows a predominance of red zones (“Highly Significant Increase”) across all Chinese waters, while blue zones (“Highly Significant Decrease”) are entirely absent. This absence is itself a key finding: it demonstrates that, unlike certain high-latitude regions where localized sea level decline is observed, China’s coastal seas are uniformly experiencing accelerated SLR with no statistically significant declining trends in any area.

As illustrated in Fig 4, the projected China SLR trends from 2030 to 2100 are derived from the integration of Sen’s slope estimator, Mann-Kendall Test, and IPCC AR6 emission scenarios. As with the global analysis, the nine-class trend classification is applied to China’s coastal seas. Notably, the map shows a predominance of red zones (“Highly Significant Increase”) across all Chinese waters, while blue zones (“Highly Significant Decrease”) are entirely absent. This absence is itself a key finding: it demonstrates that, unlike certain high-latitude regions where localized sea level decline is observed, China’s coastal seas are uniformly experiencing accelerated SLR with no statistically significant declining trends in any area.

The accelerated SLR in China’s coastal waters exhibits distinct regional patterns that reflect the influence of local oceanic and atmospheric processes. In the South China Sea, the intensification of the monsoon system under global warming alters wind stress curl and upper-ocean circulation, leading to enhanced thermal expansion and regional sea level rise [35]. The basin-scale warming of the South China Sea, coupled with its semi-enclosed configuration, amplifies steric SLR beyond the global average.

In the East China Sea, the dynamics are shaped by the interplay between the Kuroshio Current and freshwater discharge from major rivers. The Kuroshio, as the western boundary current of the North Pacific, transports warm tropical waters poleward; its strengthening and poleward shift in recent decades has contributed to accelerated warming and sea level rise in the East China Sea [29]. Concurrently, the massive freshwater and sediment input from the Yangtze River creates a distinct salinity gradient that modulates coastal density structure and steric sea level patterns [36]. The reduction in sediment supply due to upstream dam construction further exacerbates relative SLR by limiting natural land accretion [37].

These regional drivers operate against the backdrop of global thermodynamic forcing, which ensures the overall rising trend, but they introduce significant spatial heterogeneity in the magnitude and rate of SLR across China’s coastal seas [38]. The transition of previously non-significant areas in the South and East China Seas to hotspot status between 2050 and 2070 underscores the growing influence of these regional processes over time.

#### 3.1.3 Shanghai-scale analysis of results.

**Fig 5:**
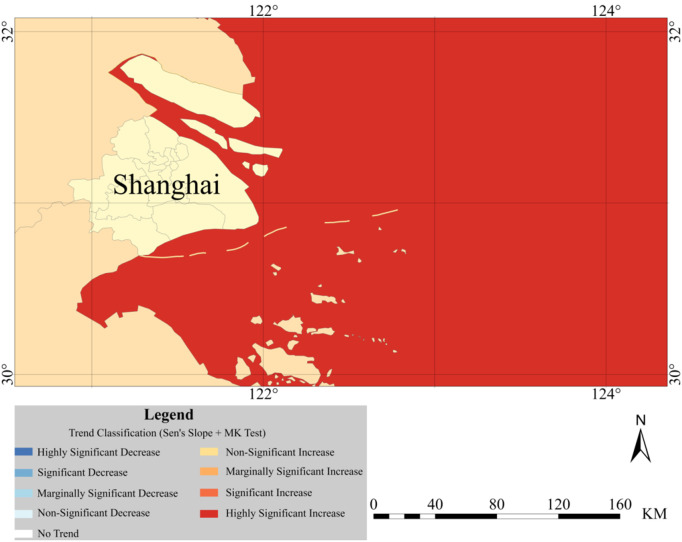
Shanghai SLR Trend (2030–2100). Maps from the Ministry of Natural Resources of the People’s Republic of China, approval number is HuS (2025) 054.

As illustrated in Fig 5, the projected Shanghai SLR trends from 2030 to 2100 are derived from the integration of Sen’s slope estimator, Mann-Kendall (M-K) Test, and IPCC AR6 (Sixth Assessment Report) emission scenarios. At the urban scale, Shanghai’s coastal waters are uniformly classified as red zones (“Highly Significant Increase”), with no other trend categories present. This homogeneity, while visually less diverse than the global or regional maps, carries important implications: it indicates that every administrative district along Shanghai’s coastline faces statistically significant SLR exposure, with no areas exhibiting stable or declining trends. The absence of other categories thus underscores the city’s uniform vulnerability to accelerated SLR.

As illustrated in Fig 5, the coastal waters of Shanghai are entirely classified as red zones, indicating highly significant rising trends in sea level with robust statistical confidence. This spatial homogeneity reveals that all administrative districts-including Jinshan, Baoshan, Pudong New Area, Yangpu, Fengxian, and Chongming-face acute exposure to accelerated SLR [39]. The uniform distribution of extreme trends aligns with regional drivers: rapid land subsidence, intensified thermal expansion in the East China Sea, and reduced sediment supply from the Yangtze River Delta due to upstream dam construction [40]. These compound pressures amplify relative SLR rates.

### 3.2. Spatial evolution of SLR

#### 3.2.1. Global-scale analysis of results.

**Fig 6:**
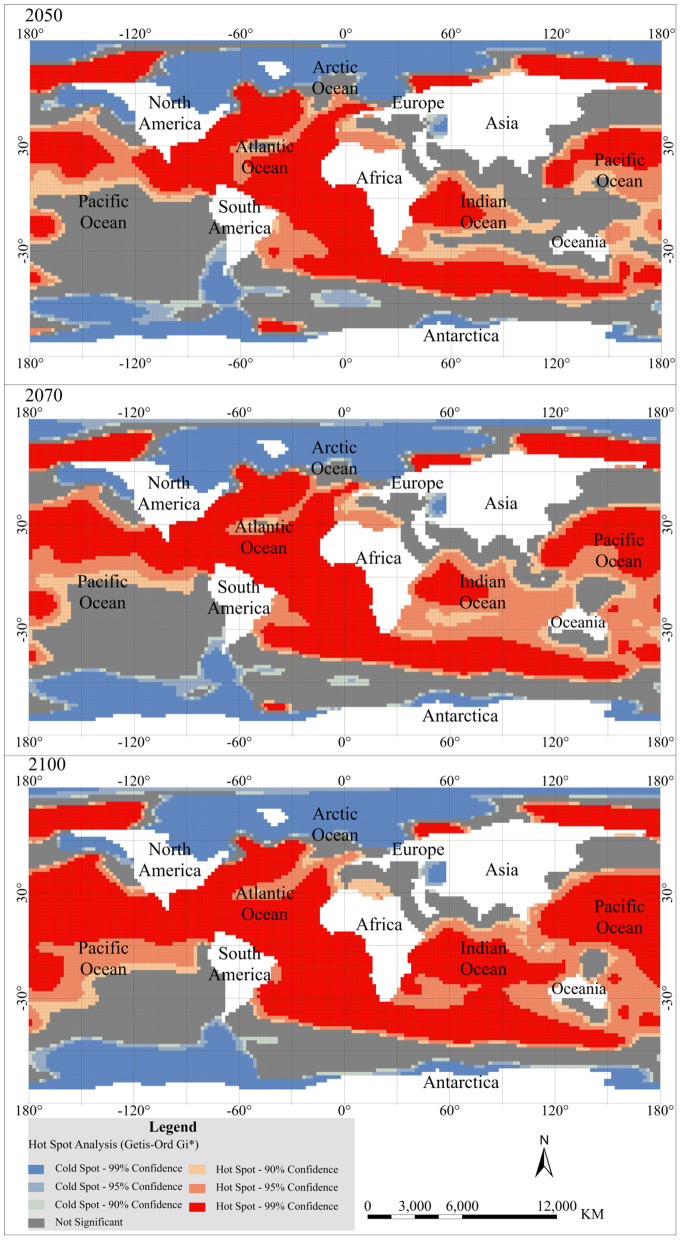
Global SLR Hotspot Map. Maps from the Ministry of Natural Resources of the People’s Republic of China, approval number is GS (2016) 2948.

The spatial autocorrelation patterns of global SLR, derived from hotspot analysis using the Getis-Ord Gi* statistic, are presented below. While Fig 6 presents static snapshots of hotspot distributions at three time points, the dynamic evolution of these patterns can be inferred by comparing the maps. Between 2050 and 2070, previously non-significant areas such as the central Pacific and central-eastern Indian Ocean transition into statistically significant hotspots, signaling escalating SLR risks. By 2070–2100, this expansion continues, with hotspots becoming more spatially coherent and persistent. The coldspots in polar regions remain relatively stable throughout the period, though their spatial extent may contract slightly as global warming intensifies.

In this study, non-significant grey points represent areas with SLR rates close to the global mean. Red points (hotspots) indicate regions where SLR rates significantly exceed the mean, while blue points (coldspots) denote areas with SLR rates significantly below the mean. The clustering of hotspots identifies priority risk zones requiring enhanced monitoring and targeted interventions.

As shown in Fig 6, SLR hotspots are predominantly located in the North Atlantic, North Indian Ocean, and North Pacific Ocean. In the North Atlantic, the North Atlantic Current enhances oceanic heat transport, driving thermal expansion and accelerating local SLR. Monsoon systems and oceanic circulation in the North Indian and Pacific Oceans alter temperature-salinity profiles, further elevating regional sea levels. Additionally, freshwater discharge from major rivers such as the Ganges and Brahmaputra modulates coastal SLR patterns through halosteric effects.

SLR coldspots are concentrated in the Arctic Ocean and Antarctic coastal zones, reflecting the spatial heterogeneity of global SLR. The Arctic’s extensive ice cover partially buffers SLR despite ongoing melt. Cold freshwater influx from ice melt temporarily suppresses thermal expansion in adjacent seas. In Antarctica, ice sheet dynamics exhibit dual effects: while ice loss contributes to global SLR, the ice sheet’s gravitational attraction and glacial isostatic adjustment counteract local SLR. Specifically, ice mass loss reduces gravitational pull on surrounding seawater, while crustal uplift from post-glacial rebound further lowers relative sea levels.

From 2050 to 2100, previously non-significant areas such as the central Pacific and central-eastern Indian Ocean transition into statistically significant hotspots, signaling escalating SLR risks. These regions are particularly sensitive to climate modes such as the El Niño-Southern Oscillation, which amplify SLR rates above the global mean through altered wind stress and oceanic heat redistribution. By 2070, spatial clustering of SLR trends is projected to intensify, with persistent or expanding hotspots underscoring the need for adaptive strategies grounded in long-term monitoring and dynamic risk assessment.

#### 3.2.2. China-scale analysis of results.

The spatial autocorrelation patterns of China SLR, derived from hotspot analysis using the Getis-Ord Gi* statistic, are presented below.

**Fig 7:**
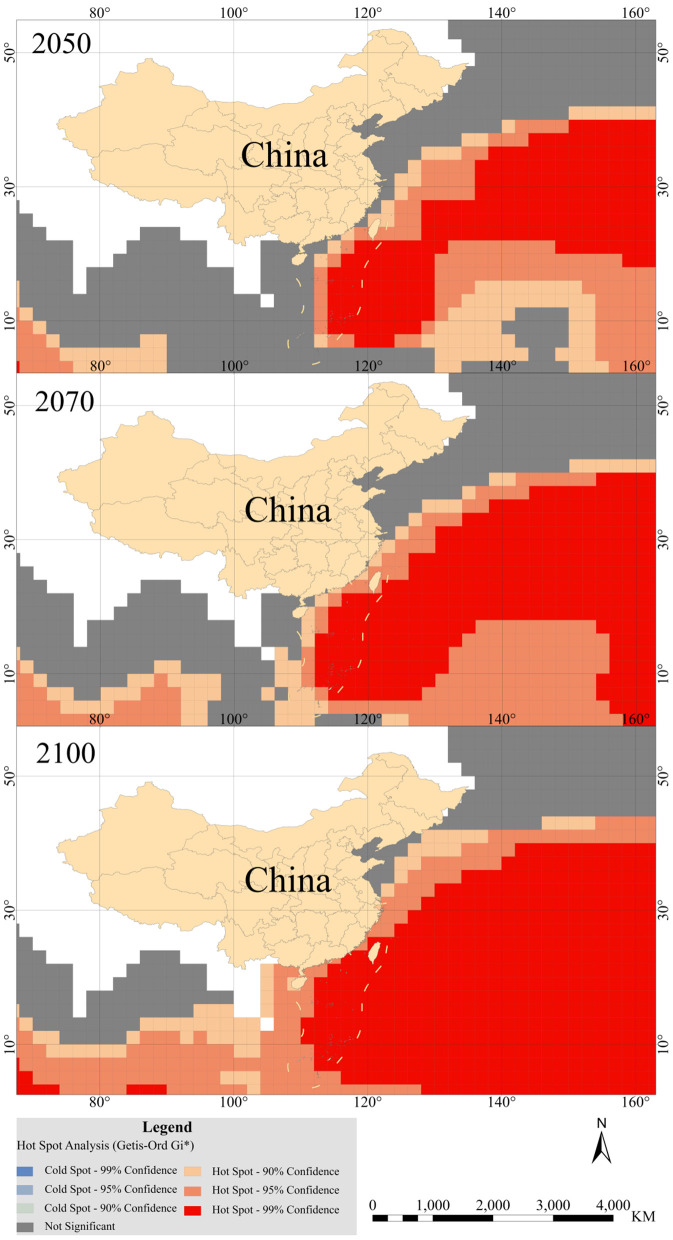
China SLR Hotspot Map. Maps from the Ministry of Natural Resources of the People’s Republic of China, approval number is GS (2016) 1600.

As shown in Fig 7, by 2100, SLR hotspots are projected in parts of China’s coastal seas, notably the South China Sea and East China Sea, where SLR rates exceed the national average. The South China Sea is influenced by multiple oceanic circulation systems, including monsoon-driven currents and branches of the Kuroshio Current (also known as the Japan Current). Variability in these circulations may alter seawater temperature and salinity, driving thermal expansion and regional SLR. Additionally, tectonic activities such as submarine volcanism and seismic events in the South China Sea could modify seabed topography, indirectly affecting local sea level through changes in water volume.

In the East China Sea, the Kuroshio Current interacts with freshwater influx from major rivers like the Yangtze and Yellow River. Sediment and freshwater inputs from these rivers significantly influence coastal salinity and density gradients, further modulating SLR patterns.

Fig 7 shows no statistically significant coldspots in Chinese waters, indicating that SLR rates across China’s coastal zones are consistently higher than the global mean. This trend is exacerbated by land subsidence caused by geological processes and human activities (e.g., groundwater extraction, urban development), which amplifies the relative SLR experienced by coastal communities. Oceanographic dynamics, including the Kuroshio Current, Taiwan Warm Current, and tidal forces, further accelerate SLR in localized areas.

Comparing hotspot maps between 2050 and 2070, a clear spatial evolution emerges: regions such as the East China Sea and South China Sea transition from non-significant to hotspot status, reflecting accelerated SLR within two decades. This shift underscores the spatiotemporal heterogeneity of SLR, driven by interactions between local factors (e.g., subsidence, riverine inputs, oceanic circulation shifts) and global climate change. For instance, increased sediment discharge from rivers or altered circulation patterns may amplify regional SLR rates, even as global thermodynamic forcing remains the primary driver.

#### 3.2.3. Shanghai-scale analysis of results.

**Fig 8:**
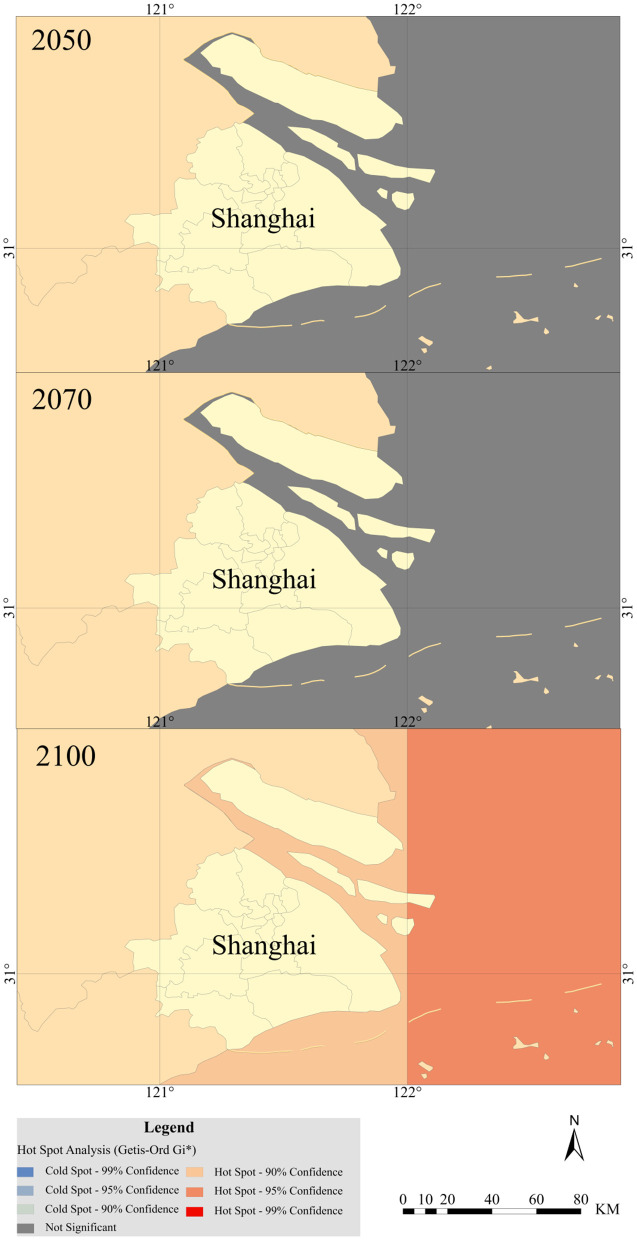
Shanghai SLR Hotspot Map. Maps from the Ministry of Natural Resources of the People’s Republic of China, approval number is HuS (2025) 054.

The spatial autocorrelation patterns of Shanghai SLR, derived from hotspot analysis using the Getis-Ord Gi* statistic, are presented in Fig 8.

By comparing hotspot maps for 2050, 2070, and 2100, we observe temporal and spatial shifts in SLR trends. Along Shanghai’s coastline, most areas are classified as either non-significant points or hotspots, indicating that local SLR rates generally match or exceed the global average. Shanghai’s location in the Yangtze River Delta-a region with complex geological settings and pronounced land subsidence-amplifies relative SLR. Subsidence, driven by groundwater extraction and urban infrastructure loads, exacerbates the perceived rise in sea level, potentially surpassing global mean rates.

Dynamic factors such as Yangtze River discharge, tidal forces, and oceanic circulation further modulate coastal SLR patterns. These processes interact across varying spatiotemporal scales to intensify local sea level changes. Notably, the hotspot maps reveal a critical transition: areas southeast of Shanghai (e.g., the East China Sea) evolve from non-significant zones in 2050 to hotspots by 2070 and 2100, signaling accelerated SLR rates in these regions over time.

## 4. Discussion

To contextualize the urban-scale findings and disentangle the multi-layered drivers of SLR, this study adopted a tri-scale analytical framework. The global-scale analysis establishes the baseline thermodynamic trend and identifies planetary-scale hotspots and coldspots driven by climate forcing and ocean circulation. The regional-scale analysis of China’s coastal seas acts as a crucial intermediary, revealing how large-scale climate modes (e.g., monsoon systems) and ocean currents (e.g., the Kuroshio) modulate the global signal, creating a distinct regional envelope of accelerated SLR. Finally, the city-scale analysis of Shanghai zooms into this regional envelope to examine how local factors—namely land subsidence, estuarine hydrodynamics, and anthropogenic modifications—further amplify and spatially structure the SLR signal. This cascading framework allows us to partition the relative contributions of global, regional, and local drivers, moving beyond a descriptive account of SLR towards a mechanistic understanding of its spatiotemporal evolution at the vulnerable urban scale.

### 4.1. Spatiotemporal evolution and drivers of global-scale SLR

Global-scale results reveal widespread and significant SLR, consistent with findings from the IPCC AR6 Report and Church et al. (2013) [8]. Using the Sen’s slope estimator and Mann-Kendall test, this study identifies highly significant SLR trends in the Atlantic, Indian, and Pacific Oceans. These trends are driven by thermal expansion (due to ocean warming) and glacial melt, which are widely recognized as primary contributors to global SLR [41]. Quantitative analyses confirm regional differences in the relative contributions of thermal expansion versus ice melt, underpinning the spatiotemporal heterogeneity of SLR. Notably, localized “highly significant declines” in high-latitude regions align with studies on glacial isostatic adjustment (GIA), highlighting the interplay between ice dynamics, gravitational effects, and local oceanographic processes [42]. Such reversals emphasize the need to examine localized physical mechanisms alongside global trends.

### 4.2. Regional characteristics of SLR in China’s coastal seas

At the national scale, China’s coastal seas exhibit highly significant SLR, corroborating high-resolution digital elevation models. China’s 18,000-kilometer coastline is shaped by complex oceanic dynamics. In the South China Sea and East China Sea, monsoon-driven currents, the Kuroshio Current, and freshwater-sediment input from the Yangtze and Yellow Rivers alter temperature-salinity profiles, intensifying thermal expansion [43]. Land subsidence-driven by coastal urbanization and groundwater extraction—aggravates relative SLR rates in densely populated areas like China. This aligns with discussions on “relative SLR”, where regional factors amplify global trends, underscoring the urgency for localized risk management [44,45].

### 4.3. Urban-scale SLR: A case study of Shanghai

The city-scale case study of Shanghai provides a granular regional perspective on SLR impacts. Results indicate that Shanghai’s urban and coastal zones universally exhibit highly significant SLR trends, with acceleration observed in localized areas. This phenomenon not only mirrors the global SLR trajectory but also underscores the profound influence of local environmental drivers. Situated in the Yangtze River Delta—a region characterized by complex geological structures and chronic land subsidence—Shanghai’s coastal SLR rates surpass both global and national averages, as evidenced by comparative hotspot maps across decades [46].

Similar to findings by Yin et al. (2013), Shanghai’s SLR dynamics are governed by multiple factors: thermal expansion, glacial melt, estuarine hydrodynamics, tidal fluctuations, and anthropogenic activities (e.g., groundwater extraction, land reclamation). This multifactorial interplay reveals intrinsic linkages between urban environmental vulnerability and global climate change, offering empirical support for adaptive coastal management [47].

Furthermore, the observed spatiotemporal clustering of SLR hotspots in Shanghai reflects complex couplings between local hydrodynamic processes (e.g., river-tide interactions) and urban development pressures [48]. These findings emphasize the necessity of integrating local geological and climatic characteristics into future urban planning and risk mitigation frameworks to address compounding SLR risks.

### 4.4. Multiscale spatiotemporal dynamics and spatial clustering

Through the integration of time-series analysis and hotspot detection, this study reveals the spatiotemporal dynamics and spatial clustering of SLR across multiple scales. From global to regional and urban levels, SLR exhibits not only accelerating trends but also pronounced spatial aggregation. Globally, hotspots cluster in the North Atlantic, Indian Ocean, and North Pacific, while in China’s coastal seas, hotspots dominate the South China Sea and East China Sea, consistent with studies on oceanic circulation and regional climate [49].

At the urban scale, Shanghai’s analysis demonstrates intensified spatial clustering of SLR due to compounding effects of land subsidence, river discharge, and local hydrological changes. This multiscale comparison highlights the spatial heterogeneity of SLR and underscores the regulatory mechanisms linking climate change, ocean dynamics, and regional geological conditions [50]. By contextualizing findings within existing research, this study emphasizes that SLR under global warming is not a linear process but a complex system shaped by multiple physical processes and regional environmental factors [51]. The intrinsic mechanisms lie in the superposition of drivers across scales and the prominence of regional characteristics. Understanding these multiscale, multifactorial coupling mechanisms is critical for refining climate models and providing scientific foundations for coastal disaster prevention and mitigation [51–55].

The three dimensions of analysis, building on existing research, fundamentally unravel the intrinsic nature and spatiotemporal evolution of SLR. The uniform upward trend at the global scale and localized heterogeneity collectively define the contemporary SLR landscape. Variations across scales arise not only from fundamental physical mechanisms (e.g., thermal expansion, glacial melt) but also reflect profound influences of regional ocean dynamics, geological subsidence, and anthropogenic activities. Such multilayered and multidimensional discourse enriches scientific understanding of SLR and provides a theoretical foundation for future higher-resolution risk assessments and adaptive strategies.

## 5. Conclusions

Adopting a cascading multi-scale analytical framework—from global and regional to city scales—this study systematically reveals the spatiotemporal evolution of SLR under climate change. By first establishing the global thermodynamic baseline and then tracing its modulation by regional ocean-atmosphere processes, we are able to isolate and quantify the critical role of local drivers in shaping the acute SLR risks faced by the megacity of Shanghai. The key findings are summarized as follows:

### 5.1. Universality and drivers of global SLR

Global sea levels exhibit widespread and significant rising trends, primarily driven by thermal expansion and ongoing melt of glaciers and polar ice sheets. These conclusions align with the IPCC AR6 Report and prior studies, confirming the decisive role of global warming in SLR. Short-term localized declines in high-latitude regions reflect complex interactions between glacial dynamics and regional oceanographic processes.

### 5.2. Regional characteristics and elevated risks in China’s coastal seas

At the national scale, China’s coastal seas demonstrate marked SLR acceleration. Regions such as the South China Sea and East China Sea show rates exceeding the global average due to monsoon circulation, Kuroshio Current dynamics, and freshwater input from major rivers (e.g., Yangtze and Yellow Rivers). Compounding effects from land subsidence (driven by natural and anthropogenic factors) further amplify relative SLR risks. These results underscore the urgent need for policy interventions to address coastal vulnerabilities.

### 5.3. Urban-scale SLR dynamics: Insights from Shanghai

The case study of Shanghai reveals pronounced SLR trends across its coastline and adjacent waters. The combined impacts of land subsidence and estuarine hydrodynamics drive relative SLR rates above global and regional averages, with distinct spatiotemporal clustering. These findings highlight the profound influence of climate change on urban environments and emphasize the need for location-specific adaptation measures in infrastructure planning and risk management.

### 5.4. Multiscale spatiotemporal dynamics and clustering mechanisms

Comparative analyses across global, regional, and urban scales demonstrate accelerating SLR trends with significant spatial clustering. Global hotspots cluster in the North Atlantic, Indian, and Pacific Oceans, while China’s hotspots concentrate in the South and East China Seas. At the city scale, Shanghai’s SLR patterns reflect localized feedbacks between geological subsidence, river-tide interactions, and oceanic processes. Such multiscale heterogeneity arises from the coupling of global warming, regional ocean dynamics, and local environmental conditions, providing a systemic framework to unravel SLR mechanisms.

In summary, global SLR is both a critical indicator of climate change and a complex, multiscale system shaped by intertwined global and local factors. The coexistence of uniform global trends and regional heterogeneity defines the current and future trajectory of SLR. These findings offer robust scientific support for coastal risk assessment, disaster mitigation, and adaptive urban planning, while guiding future research to refine SLR projections and explore localized physical mechanisms.
