# Supplementary material for: Rising tides: Unveiling the spatial and temporal evolution of sea level rise under climate change
Source: PLoS One. 2026 Jun 17;21(6):e0347855. doi: 10.1371/journal.pone.0347855 (PMC13274844; doi:10.1371/journal.pone.0347855)
Supplement: Attachment — Submitted filename: Response to Reviewers.pdf [file pone.0347855.s002.pdf]

Journal Requirements (2) :

1. Thank you for updating your data availability statement. You note that your data are available within the Supporting Information files, but no such files have been included with your submission. At this time we ask that you please upload your minimal data set as a Supporting Information file, or to a public repository such as Figshare or Dryad.

Please also ensure that when you upload your file you include separate captions for your supplementary files at the end of your manuscript.

As soon as you confirm the location of the data underlying your findings, we will be able to proceed with the review of your submission.

Response： Thank you for your comment,we have added Data Availability Statement in the manuscript: All relevant data are within the manuscript and its Supporting Information files. Data Registration DOI: https://doi.org/10.17605/OSF.IO/V6DRJ.

2. Thank you for providing this information on where your map images in Figures 2, 3, 4, 5, 6, 7, and 8 were from. We need one further update to these figures before we can proceed. Maps from the Ministry of Natural Resources of the People's Republic of China need to have the approval number/ID for the map included in the figure captions. Could you please update your figure captions to include this information?

Response： Thank you for your comment,we have added the approval number.

Journal Requirements (1) :

When submitting your revision, we need you to address these additional requirements.

1. Please ensure that your manuscript meets PLOS ONE's style requirements, including those for file naming. The PLOS ONE style templates can be found at

https://journals.plos.org/plosone/s/file?id=wjVg/PLOSOne_formatting_sample_main_body.pdf and

https://journals.plos.org/plosone/s/file?id=ba62/PLOSOne_formatting_sample_title_authors_affiliations.pdf

Response： Thank you for your comment, We are ensured that our manuscript meets PLOS ONE's style requirements.

2. Please note that PLOS One has specific guidelines on code sharing for submissions in which author-generated code underpins the findings in the manuscript. In these cases, we expect all author-generated code to be made available without restrictions upon publication of the work. Please review our guidelines at https://journals.plos.org/plosone/s/materials-and-software-

sharing#loc-sharing-code and ensure that your code is shared in a way that follows best practice and facilitates reproducibility and reuse.

Response： Thank you for your comment, this manuscript does not involve any author-generated code.

3. We note that the grant information you provided in the ‘Funding Information’ and ‘Financial Disclosure’ sections do not match.

When you resubmit, please ensure that you provide the correct grant numbers for the awards you received for your study in the ‘Funding Information’ section.

Response： Thank you for your comment, We have identified the following two fund projects in the manuscript: this research was supported by the National Social Science Fund of China （23CXW034） ; and The Key Research Project of the National Foundation of Social Science of China: Community Governance and Post-relocation Support in Cross District Resettlement [grant number 21&ZD183].

4. Thank you for stating the following financial disclosure:
This research was supported by the National Social Science Fund of China （23CXW034） ;& Fundamental Research Funds for the Central Universities: Climate Migration Types and Risk Management in Coastal Areas. (grant number B230205032); &Postgraduate Research & Practice Innovation Program of Jiangsu Province: Climate Migration Types and Risk Management in Coastal Areas. (grant number 422003151); and The Key Research Project of the National Foundation of Social Science of China: Community Governance and Post-relocation Support in Cross District Resettlement [grant number 21&ZD183].

Please state what role the funders took in the study. If the funders had no role, please state: "The funders had no role in study design, data collection and analysis, decision to publish, or preparation of the manuscript."
If this statement is not correct you must amend it as needed.
Please include this amended Role of Funder statement in your cover letter; we will change the online submission form on your behalf.

Response： Thank you for your comment, we have stated the sentence in cover letter, which is "The funders had no role in study design, data collection and analysis, decision to publish, or preparation of the manuscript."

5. We note that your Data Availability Statement is currently as follows: All relevant data are within the manuscript and its Supporting Information files. Data Registration DOI: https://doi.org/10.17605/OSF.IO/V6DRJ.

Please confirm at this time whether or not your submission contains all raw data required to replicate the results of your study. Authors must share the "minimal data set" for their submission. PLOS defines the minimal data set to consist of the data required to replicate all study findings reported in the article, as well as related metadata and methods (https://journals.plos.org/plosone/s/data-availability#loc-minimal-data-set-definition).

For example, authors should submit the following data:

- The values behind the means, standard deviations and other measures reported;

- The values used to build graphs;

- The points extracted from images for analysis.

Authors do not need to submit their entire data set if only a portion of the data was used in the reported study.

If your submission does not contain these data, please either upload them as Supporting Information files or deposit them to a stable, public repository and provide us with the relevant URLs, DOIs, or accession numbers. For a list of recommended repositories, please see https://journals.plos.org/plosone/s/recommended-repositories.

If there are ethical or legal restrictions on sharing a de-identified data set, please explain them in detail (e.g., data contain potentially sensitive information, data are owned by a third-party organization, etc.) and who has imposed them (e.g., an ethics committee). Please also provide contact information for a data access committee, ethics committee, or other institutional body to which data requests may be sent. If data are owned by a third party, please indicate how others may request data access.

Response： Thank you for your comment, we have confirmed that at this time our submission contains all raw data required to replicate the results of our study.

6. We note that Figures 2, 3, 4, 5, 6, 7, and 8 in your submission contain map images which may be copyrighted. All PLOS content is published under the Creative Commons Attribution License (CC BY 4.0), which means that the manuscript, images, and Supporting Information files will be freely available online, and any third party is permitted to access, download, copy, distribute, and use these materials in any way, even commercially, with proper attribution. For these reasons, we cannot publish previously copyrighted maps or satellite images created using proprietary data, such as Google software (Google Maps, Street View, and Earth). For more information, see our copyright guidelines: http://journals.plos.org/plosone/s/licenses-and-copyright.

We require you to either (a) present written permission from the copyright holder to publish these figures specifically under the CC BY 4.0 license, or (b) remove the figures from your submission:

a. You may seek permission from the original copyright holder of Figures 2, 3, 4, 5, 6, 7, and 8 to publish the content specifically under the CC BY 4.0 license.

We recommend that you contact the original copyright holder with the Content Permission Form (http://journals.plos.org/plosone/s/file?id=7c09/content-permission-form.pdf) and the following text:

“I request permission for the open-access journal PLOS ONE to publish XXX under the Creative Commons Attribution License (CCAL) CC BY 4.0 (http://creativecommons.org/licenses/by/4.0/). Please be aware that this license allows unrestricted use and distribution, even commercially, by third parties. Please reply and provide explicit written permission to publish XXX under a CC BY license and complete the attached form.”

Please upload the completed Content Permission Form or other proof of granted permissions as an ""Other"" file with your submission.

In the figure caption of the copyrighted figure, please include the following text: “Reprinted from [ref] under a CC BY license, with permission from [name of publisher], original copyright [original copyright year].”

b. If you are unable to obtain permission from the original copyright holder to publish these figures under the CC BY 4.0 license or if the copyright holder’s requirements are incompatible with the CC BY 4.0 license, please either i) remove the figure or ii) supply a replacement figure that complies with the CC BY 4.0 license. Please check copyright information on all replacement figures and update the figure caption with source information. If applicable, please specify in the figure caption text when a figure is similar but not identical to the original image and is therefore for illustrative purposes only.

The following resources for replacing copyrighted map figures may be helpful:

USGS National Map Viewer (public domain): http://viewer.nationalmap.gov/viewer/

The Gateway to Astronaut Photography of Earth (public domain): http://eol.jsc.nasa.gov/sseop/clickmap/

Maps at the CIA (public domain): https://www.cia.gov/library/publications/the-world-factbook/index.html and https://www.cia.gov/library/publications/cia-maps-publications/index.html

NASA Earth Observatory (public domain): http://earthobservatory.nasa.gov/

Landsat: http://landsat.visibleearth.nasa.gov/

USGS EROS (Earth Resources Observatory and Science (EROS) Center) (public domain): http://eros.usgs.gov/#

Natural Earth (public domain): http://www.naturalearthdata.com/

Response： Thank you for your comment, The map images in Figures 2, 3, 4, 5, 6, 7, and 8 of the manuscript are sourced from the website of the Ministry of Natural Resources of the People's Republic of China, available at: **http://bzdt.ch.mnr.gov.cn.**

According to the usage guidelines provided on the website, these standard maps are compiled based on the established standards for depicting the boundary lines of China and other countries worldwide. They are intended for various purposes, including news media illustrations, book and periodical graphics, advertising backgrounds, and reference designs for handicrafts. They may also serve as base maps for creating publicly available maps. The general public can freely browse and download these standard maps, as they are not subject to copyright protection.

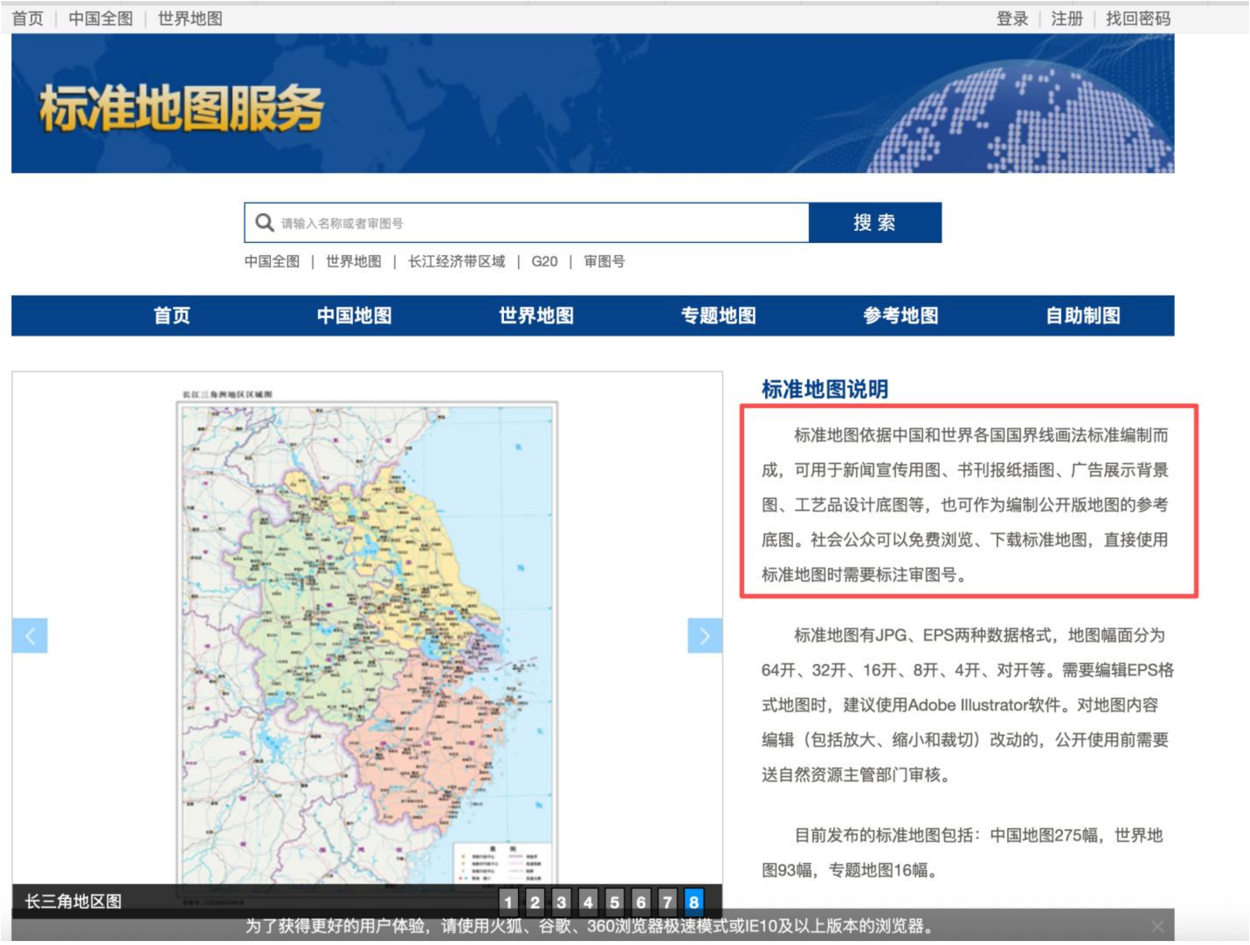

7. Please include a copy of Table 1 which you refer to in your text on page 8.

Response： Thank you for your comment, Table 2 in the manuscript is actually Table 1; it was a writing error. We have now corrected this and updated the table reference accordingly in the revised manuscript.

8. We note you have included a table to which you do not refer in the text of your manuscript. Please ensure that you refer to Table 2 in your text; if accepted, production will need this reference to link the reader to the Table.

Response： Thank you for your comment, Table 2 in the manuscript is actually Table 1; it was a writing error. We have now corrected this and updated the table reference accordingly in the revised manuscript.

9. If the reviewer comments include a recommendation to cite specific previously published works, please review and evaluate these publications to determine whether they are relevant and should be cited. There is no requirement to cite these works unless the editor has indicated otherwise.

Response： Thank you for your comment, We followed this comment.

Reviewer #1

In general, the manuscript is well written, and it is easy to understand for an international reader. Th structure of the paper is good, and the figures have good visual quality. The references used in this study represents high standard international studies and supports well the text. Despite the good quality of the grammar, the text should be edited to remove (or add spaces) between the text and sone numbers of the figures (line 219 as example).

Response： Thank you for your comment, the spaces in the manuscript have been corrected.

The introduction presents what it's known about SLR values and possible impacts and the drivers of sea level rise at different spatial scales, linking the process with thermal expansion and the melting of ice caps, following a brief description of SLR. Then, a description of what is spatiotemporal evolution and why its study is important.

Response： Thank you for your comment, The fourth paragraph of the introduction has already provided relevant descriptions, as follows:Under this context, elucidating the Spatiotemporal Evolution characteristics of SLR carries significant scientific and practical implications. The term "Spatiotemporal Evolution" refers to the dynamic variation process of SLR across temporal sequences and spatial distributions[16]. This conceptual framework emphasizes that SLR is not a globally uniform phenomenon, but rather a complex process shaped by the interplay of regional climate patterns, ocean dynamics, and terrestrial surface processes[17]. Comprehending the Spatiotemporal Evolution of SLR enables us to not only decipher its historical variation patterns and future trajectories, but also provides crucial foundations for developing targeted adaptation and mitigation strategies[18]. Practical applications include: identifying SLR high-risk zones to prioritize flood defense infrastructure deployment and population relocation planning; analyzing its temporal evolution patterns to forecast both near-term and long-term risks, thereby informing critical infrastructure design and land-use planning decisions[19].

The authors refer that there are few studies with high resolution results at small scale. However, in the literature there are many examples of the impact of SLR in coastal systems (in variables like salinity intrusion, inundation areas, biogeochemistry, etc), like coastal seas and estuaries throughout the world and, in my opinion, you can rewrite the introduction to present those studies.

Response： Thank you for your comment, The fifth paragraph of the introduction has already provided relevant descriptions, as follows:However, a critical gap persists in the comprehensive understanding of SLR's spatiotemporal evolution, particularly regarding its high-resolution characterization at local scales. A rich body of literature has extensively documented the impacts of SLR on coastal systems, such as salinity intrusion, inundation extent, and biogeochemical cycles in estuaries and coastal seas worldwide [20-21]. These studies are crucial for assessing vulnerability and informing adaptation. Nevertheless, the vast majority of this research treats SLR as a boundary condition—often derived from coarse global or regional models—rather than focusing on the SLR signal itself as a dynamic, spatially heterogeneous process. Consequently, there is a distinct lack of high-resolution analysis that disentangles the localized spatiotemporal evolution of SLR in critical areas like megacities or estuarine zones [22]. For instance, in coastal megacities such as Shanghai, where land subsidence and complex estuarine hydrodynamics interact to amplify local SLR characteristics well beyond global averages, detailed investigations into the precise spatiotemporal patterns of this amplified rise remain insufficient [23].

The main objectives are well presented and described. The focus is the local scale, and it is not clear why the authors choose to do the analysis in global and regional scales and another aspect that is not clear is the link of the global and regional scales to the local scale analysis. You should improve this presentation.

Response： Thank you for your comment, We have added relevant statements in section 4 discussion , as follows:To contextualize the urban-scale findings and disentangle the multi-layered drivers of SLR, this study adopted a tri-scale analytical framework. The global-scale analysis establishes the baseline thermodynamic trend and identifies planetary-scale hotspots and coldspots driven by climate forcing and ocean circulation. The regional-scale analysis of China's coastal seas acts as a crucial intermediary, revealing how large-scale climate modes (e.g., monsoon systems) and ocean currents (e.g., the Kuroshio) modulate the global signal, creating a distinct regional envelope of accelerated SLR. Finally, the city-scale analysis of Shanghai zooms into this regional envelope to examine how local factors—namely land subsidence, estuarine hydrodynamics, and anthropogenic modifications—further amplify and spatially structure the sea level rise signal. This cascading framework allows us to partition the relative contributions of global, regional, and local drivers, moving beyond a descriptive account of SLR towards a mechanistic understanding of its spatiotemporal evolution at the vulnerable urban scale.

We also have added relevant statements in section 5 Conclusions , as follows:Adopting a cascading multi-scale analytical framework—from global and regional to city scales—this study systematically reveals the spatiotemporal evolution of SLR under climate change. By first establishing the global thermodynamic baseline and then tracing its modulation by regional ocean-atmosphere processes, we are able to isolate and quantify the critical role of local drivers in shaping the acute SLR risks faced by the megacity of Shanghai. The key findings are summarized as follows:

The study area section presents a brief description of the local area of Shanghai. The description of the study region is made poorly. Loca elevation and maximum precipitation are described but no information regarding tidal amplitude is provided. Moreover, rivers are referred to be important, but no information about freshwater inflow values and its importance to local trends is

given. More details should be included to improve this section (Study Area), which will be useful to international readers.

Response： Thank you for your comment, We have rewritten the 2.1 Study Area as follows:

Shanghai is situated on the eastern coast of China at the southern mouth of the Yangtze River, forming the core of the Yangtze River Delta (YRD) alluvial plain (Figure 2). Its geographical location makes it one of the most prominent megacities globally that is acutely vulnerable to climate-induced sea level rise .

The city's topography is characteristically low and flat, with an average elevation of approximately 4 meters above the mean sea level, and large portions of the urban area lying less than 3.5 meters above sea level. This low-lying topography is a product of its deltaic origins, composed of unconsolidated Holocene sediments that are naturally prone to compaction.

Shanghai experiences a northern subtropical monsoon climate, characterized by distinct seasons and abundant rainfall, with an annual precipitation averaging 1,200 mm. Critically, the local hydrodynamic regime is dominated by a regular semi-diurnal tide. The mean tidal range at the mouth of the Yangtze River is approximately 2.4 to 2.8 meters, with a maximum spring tidal range that can exceed 4.5 meters. This significant tidal amplitude, combined with the funnel-shaped estuary, amplifies storm surges and facilitates the upstream propagation of tidal waves, directly influencing water levels in the Huangpu River that flows through the city center.

The Yangtze River, the longest river in Asia, discharges an enormous volume of freshwater and sediment into the East China Sea via Shanghai. Its multi-annual mean freshwater discharge is approximately 900 billion cubic meters per year, with a sediment load that historically exceeded 400 million tons annually. This massive freshwater inflow creates a distinct salinity gradient in the coastal waters, influencing density-driven circulation and, consequently, local steric sea level patterns. However, the construction of major upstream dams, most notably the Three Gorges Dam, has drastically reduced the sediment supply to the delta by over 70% in recent decades. This sediment starvation exacerbates the relative SLR by inhibiting natural land accretion and potentially accelerating subsidence.

Furthermore, Shanghai is afflicted by significant land subsidence, a critical compounding factor for relative SLR. Driven by a century of extensive groundwater extraction for industrial and domestic use, as well as the immense load of its urban infrastructure, some areas of the city have experienced cumulative subsidence exceeding 3 meters since the 1920s. Although extraction rates have been regulated, residual compaction and ongoing urban construction continue to cause a subsidence rate of several millimeters per year in many districts.

As a global financial and transportation hub with over 24 million residents, Shanghai's low elevation, dense population, and high-value infrastructure make it a global priority case for understanding and adapting to the multiscale drivers of SLR. The city's vulnerability is not merely a function of global climate change, but is profoundly shaped by the dynamic interplay of its local tidal regime, riverine inputs, and anthropogenic subsidence.

In section 2.2. the data sources are presented. However, in the text, only the names of the data sets are given. More information about the data sources is needed. Are the data daily, monthly,

hourly? How about the spatial resolution of the data? You should refer this and then you can give the references for the data.

Response： Thank you for your comment, We have rewritten the 2.2 Data Sourcesas follows:

This study synthesizes multi-source datasets to analyze the spatiotemporal evolution of SLR across global, regional, and urban scales. The primary data sources and their characteristics are as follows:

① IPCC AR6 Sea Level Projection Dataset

The global and regional sea level projections are derived from the Sixth Assessment Report (AR6) of the Intergovernmental Panel on Climate Change (IPCC), specifically from the datasets underpinning Working Group I's Chapter 9 ("Ocean, Cryosphere, and Sea Level Change") and the Summary for Policymakers. The IPCC AR6 provides sea level projections for five Shared Socioeconomic Pathways (SSPs): SSP1-1.9, SSP1-2.6, SSP2-4.5, SSP3-7.0, and SSP5-8.5. These scenarios depict various development pathways of future economic and social systems, reflecting the correlation between societal development and the challenges of mitigating and adapting to climate change. They serve as the core foundation for conducting climate change impact assessments and climate policy formulation. In this study, we primarily use sea level projection data under the intermediate emission scenario SSP2-4.5 for analysis.

Spatial Coverage: Global ocean, with regional breakdowns including China's coastal seas.

Spatial Resolution: Data are provided on a 1° × 1° latitude-longitude grid for global fields; regional mean time series are also available for predefined coastal segments.

Temporal Coverage: 2020–2100 (with extensions to 2150 for some scenarios).

Temporal Resolution: Annual mean sea level anomalies relative to a 1995–2014 baseline.

Data Components: The dataset includes total sea level rise as well as contributions from its primary components: thermal expansion, glacier mass loss, Greenland ice sheet melt, Antarctic ice sheet melt, and land water storage changes.

② NASA Sea Level Projection Tool

To complement the IPCC AR6 data and facilitate site-specific analysis, we also utilized the NASA Sea Level Projection Tool, which visualizes and distributes the AR6 scenario outputs alongside observational tide gauge records. The data were accessed via the NASA Sea Level Change Portal.

Data Source: The tool integrates IPCC AR6 projections with NASA's observational datasets for validation. The underlying model outputs are consistent with the IPCC AR6 data described above.

Query Parameters: For the Shanghai urban-scale analysis, we extracted projections for the grid cell corresponding to the Yangtze River Estuary (approximately 31.5°N, 122.0°E).

Spatial Resolution: The tool provides data at the native 1° × 1° grid resolution of the underlying models, with the ability to extract time series for specific coordinates.

Temporal Coverage and Resolution: Same as the IPCC AR6 dataset (2020–2100, annual means).

Access: The data were retrieved from: https://sealevel.nasa.gov/ipcc-ar6-sea-level-projection-tool.

The authors used a method that uses the Theil-Sen Median estimator and claims that this methos is robust against outliers and noisy data. This is a fact, the authors are right, but the question here is: Why use it with SLR data? These data present outliers and a noisy temporal evolution? Despite this question, the methods are very well described.

Response: Thank you for your comment, You are absolutely correct that simply stating the method is "robust against outliers" is insufficient without justifying its necessity for our specific dataset. We have now added a detailed rationale in Section 2.3.1 explaining why SLR projection data, despite having a strong long-term trend, contain inherent variability from sources such as ENSO, PDO, and regional ocean-atmosphere processes. This interannual-to-decadal variability can act as "noise" that may bias ordinary least squares trend estimates. The Theil-Sen estimator, by employing the median of pairwise slopes, effectively mitigates the influence of these short-term fluctuations, ensuring that our identified trends (Figure 3-5) represent robust long-term signals rather than artifacts of transient climate variability. We believe this clarification strengthens the methodological justification and addresses your concern.

In the results section, the authors tell us that the IPCC AR6 emission scenario are used. You should clearly present the emission scenarios that were used in this study. You should introduce these details in section 2.2.

Response: Thank you for your comment, We have provided additional explanations in section 2.2.

The description of the results can be improved.in lines 219-221, the authors refer that the Seen's slope and the MK test categorizes the trends into nine distinct classes. I believe that, but this result is not visible in Figure 3. You should show the classes explicitly, maybe with zooms for more localizes locations.

Response: Thank you for your comment, You are correct that Figure 3 displays a legend with nine trend categories, but some of these categories (e.g., dark blue for "Highly Significant Decrease") are not visibly present in the map. This is not an oversight but rather an intentional and meaningful aspect of the visualization: the absence of certain categories itself conveys information. In the revised manuscript, we have clarified this point in multiple locations (Section 3.1.1; Section 3.1.2; Section 3.1.3) by explicitly stating which categories appear in each map and discussing the implications. For example, we now explain that the absence of declining trends in China's coastal seas (Figure 4) indicates uniform acceleration across the region, while the presence of both increases and decreases in the global map (Figure 3) highlights the

spatiotemporal heterogeneity of SLR. We believe these additions enhance the reader's ability to interpret the figures correctly and appreciate the full meaning of the trend classification system.

In the following I will include some comments about the presentation of the results. I think that this section should be rewritten because:

1) In Figure 3, 9 classes are displayed, but most of the classes (different from “Highly Significant Increase”) are not visible. To improve the display of the results, maybe some zooms should be generated near the coastal areas. This comment is valid to every figure showing different classification classes at global, regional or local scale.

Response： Thank you for your comment, We have provided additional explanations after the Mann Kendall Test table in section 2.3.1 (2), as follows:It is important to note that the nine trend categories defined in Table 1 represent the full spectrum of possible outcomes from the combined Sen's slope and MK Test analysis. However, depending on the study area and scale, not all categories may appear in the resulting maps. The presence or absence of specific categories in itself provides valuable information about the regional characteristics of SLR. For instance, the absence of declining trends (categories -1 to -4) in a given area indicates that all locations are experiencing either stable or rising sea levels, with no statistically significant decreases. Conversely, the presence of both increasing and decreasing trends within the same region would suggest complex, heterogeneous dynamics.

we have clarified this point in multiple locations (Section 3.1.1; Section 3.1.2; Section 3.1.3) by explicitly stating which categories appear in each map and discussing the implications. For example, we now explain that the absence of declining trends in China's coastal seas (Figure 4) indicates uniform acceleration across the region, while the presence of both increases and decreases in the global map (Figure 3) highlights the spatiotemporal heterogeneity of SLR. We believe these additions enhance the reader's ability to interpret the figures correctly and appreciate the full meaning of the trend classification system.

2) The results are discussed in a rather qualitative manner. Moreover, the presentation of the results from the analysis of the data relates to phenomenon’s driving SLR (e.g. glacial melting or thermal expansion of the ocean). In these parts of the text you should introduce some references. Between lines 237 and 249, there is a whole paragraph where supporting references are needed.

Response： Thank you for your comment, we have added relevant references.

3) Between lines 256 and 267, the analysis for the coast of China is presented. The comment 2) is valid for this figure. At the bottom of the page, a connection between the results and remote forcing effects is presented. However, the connection between these remote forcing and regional scale results should be supported by references to other studies where the same (or similar) class classification was described.

Response： Thank you for your comment,we have added relevant references.

4) The same is applicable to the local scale of the area of Shanghai.

Response： Thank you for your comment,we have added relevant references.

5) The authors uses in the manuscript two different reference types: the numeric and the classical last name of the author plus the yea. Please present the references in an uniform manner.

Response： Thank you for your comment, we have unified it into digital references.

6) Figure 6 depicts the results for 2050, 2070 and 2100. To a clearly presentation, the authors should consider depicting differences from 2070 and 2100 to 2050 or to another reference period. This should improve the presentation of the results.

Response： Thank you for your comment, we have provided the following supplementary explanations in section 3.2.1

While Figure 6 presents static snapshots of hotspot distributions at three time points, the dynamic evolution of these patterns can be inferred by comparing the maps. Between 2050 and 2070, previously non-significant areas such as the central Pacific and central-eastern Indian Ocean transition into statistically significant hotspots, signaling escalating SLR risks. By 2070-2100, this expansion continues, with hotspots becoming more spatially coherent and persistent. The coldspots in polar regions remain relatively stable throughout the period, though their spatial extent may contract slightly as global warming intensifies.

Reviewer #2

1. Critique of Figure 1 (Mechanism Diagram)

The current Figure 1, titled "Mechanism Diagram of SLR," is presented as a conceptual cartoon that illustrates greenhouse gas emissions, global warming, and ocean responses in a highly simplified manner. While it captures the general flow of the problem, it lacks the scientific depth and technical data expected in a research article.

Proposed Technical Diagram: To improve the manuscript's professionalism, Figure 1 should be replaced with a systems-based physical schematic. A more technical diagram should include:

• External Forcing: Explicitly labeling radiative forcing and specific Greenhouse Gases (GHGs).

• Thermodynamic Components: Differentiating between steric SLR (thermal expansion and halosteric effects) and mass-driven SLR (ice sheet discharge and glacier melt).

• Regional Modulators: Adding nodes for Glacial Isostatic Adjustment (GIA), ocean dynamic processes like the AMOC slowdown, and localized land subsidence.

• Interactions: Using technical flowlines to show how polar amplification affects seawater density and volume.

Response： Thank you for your comment, We acknowledge that the current figure is presented as a simplified conceptual diagram. However, the Introduction is primarily intended to provide a

general background for the study, not to delve into the detailed physical mechanisms of sea level rise. To better reflect this purpose, we have renamed Figure 1 from "Mechanism Diagram of SLR" to "Schematic Diagram of SLR" to avoid any overstatement of its technical depth.

2. Restructuring of the Methodology (Section 2.3)

The presentation of the Theil-Sen Median Estimator and the Mann-Kendall Test in Section 2.3 currently resembles a technical report or a manual rather than an academic paper.

Required Improvements:

• Narrative Integration: Phrases such as "Step 1: Compute the MK Statistic S" or "Step 2: Perform Trend Test" should be converted into a continuous narrative. For example: "The statistical significance of the detected trends was evaluated using the standardized Z-statistic, derived from the variance of S..."

• Equation Formatting: Equations (1-1) through (1-7) should be better integrated into the text syntax. Every variable (e.g., n, sgn, wij) must be formally defined immediately following the equation in which it first appears.

• Unification of Variables: Ensure consistent typography for variables throughout the section to avoid the appearance of a fragmented report.

Response： Thank you for your comment, In response, we have completely restructured Section 2.3.1 to achieve a more integrated narrative flow. The step-by-step instructions ("Step 1," "Step 2") have been removed and replaced with continuous prose that explains the logical progression of the analysis. Each variable (e.g., n for sample size, sgn for the sign function, wij for spatial weights) is now formally defined immediately following its first appearance in an equation, ensuring clarity for readers. We have also unified the typography of all mathematical symbols throughout the section to maintain consistency. Finally, we strengthened the connection between the methods and their justification by explicitly linking the Theil-Sen estimator's robustness properties to the inherent variability in SLR data (e.g., ENSO-driven fluctuations, regional process noise). We believe these revisions substantially improve the professionalism and readability of the methodology section. Thank you for guiding us toward this improvement.

3. Additional Technical Comments

• Abstract Terminology: The abstract uses highly abstract terms such as "epistemic hybridization" and "anticipatory governance tools". While innovative, these terms should be more clearly linked to the specific quantitative results of the tri-scale framework.

• Scale Redundancy: There is significant repetition of descriptive text between the global scale (3.1.1) and China scale (3.1.2) results. Specifically, the paragraphs explaining AMOC slowdown and halosteric effects are nearly identical in both sections. Each scale analysis should focus on its unique regional drivers rather than restating global mechanisms.

• Subsidence Attribution: The study highlights land subsidence as a major driver for Shanghai's relative SLR. The manuscript would benefit from clarifying if the NASA projection models used

already account for anthropogenic subsidence (e.g., groundwater extraction) or if this was a separate parameter integrated by the authors.

• Visualization Consistency: The maps in Figures 3, 4, and 5 use a 9-class trend classification. Providing a summary table with the specific median slope values (β) for key regions would provide the quantitative evidence necessary to support the qualitative "Hotspot" descriptions

Response： Thank you for your comment.

(1)Summary Terminology

Thank you very much for pointing out this issue. We agree that although these terms reflect the innovation of the research, they may make readers feel abstract and vague without a clear connection to specific results. We will revise the relevant statements in the abstract to directly link these concepts with the quantitative results of the three scale framework

(2)Redundancy in scale description

Section 3.1.2 does unnecessarily repeat the detailed explanation of global mechanisms (AMOC, GIA, salt reduction effects) in Section 3.1.1, which have weak direct relevance to China's offshore waters. We restructured section 3.1.2 to focus on the unique regional driving factors in China's offshore areas.

(3) Subsidence Attribution

We provided the following explanation in section 2.2 Data Sources: It is important to clarify that the IPCC AR6 and NASA sea level projection datasets used in this study provide projections of absolute sea level rise—the change in ocean surface height relative to the geocentric reference frame. These global and regional climate models do not, by design, incorporate localized anthropogenic processes such as groundwater extraction-induced subsidence, sediment compaction from urban loading, or other human-driven land surface changes.

(4) Visual consistency and quantitative evidence

This is a very practical suggestion. Indeed, relying solely on color classification charts cannot provide readers with specific trend values. We have added a summary table 2 in the 3.1 Temporary Evolution of SLR section, listing the Sen's slope median (β) and MK test Z-values for key regions, providing quantitative support for hotspot description.
