# Supplementary material for: Rising tides: Unveiling the spatial and temporal evolution of sea level rise under climate change
Source: PLoS One. 2026 Jun 17;21(6):e0347855. doi: 10.1371/journal.pone.0347855 (PMC13274844; doi:10.1371/journal.pone.0347855)
Supplement: Peer review history

# Peer review: Decision Letter 0

**DOI:** 10.1371/journal.pone.0347855.r001

**Authors:** João Miguel Dias

18 Feb 2026

PONE-D-25-55574

Rising Tides: Unveiling the Spatial and Temporal Evolution of Sea Level Rise Under Climate Change

PLOS One

Dear Dr. Liang,

Thank you for submitting your manuscript to PLOS ONE. After careful consideration, we feel that it has merit but does not fully meet PLOS ONE’s publication criteria as it currently stands. Therefore, we invite you to submit a revised version of the manuscript that fully addresses all the points raised during the review process by both reviewers.

Please submit your revised manuscript by Apr 04 2026 11:59PM. If you will need more time than this to complete your revisions, please reply to this message or contact the journal office at plosone@plos.org. When you're ready to submit your revision, log on to https://www.editorialmanager.com/pone/ and select the 'Submissions Needing Revision' folder to locate your manuscript file.. When you're ready to submit your revision, log on to https://www.editorialmanager.com/pone/ and select the 'Submissions Needing Revision' folder to locate your manuscript file.

Please include the following items when submitting your revised manuscript:

- A letter that responds to each point raised by the academic editor and reviewer(s). You should upload this letter as a separate file labeled 'Response to Reviewers'.
- A marked-up copy of your manuscript that highlights changes made to the original version. You should upload this as a separate file labeled 'Revised Manuscript with Track Changes'.
- An unmarked version of your revised paper without tracked changes. You should upload this as a separate file labeled 'Manuscript'.

If you would like to make changes to your financial disclosure, please include your updated statement in your cover letter. Guidelines for resubmitting your figure files are available below the reviewer comments at the end of this letter.

If applicable, we recommend that you deposit your laboratory protocols in protocols.io to enhance the reproducibility of your results. Protocols.io assigns your protocol its own identifier (DOI) so that it can be cited independently in the future. For instructions see: https://journals.plos.org/plosone/s/submission-guidelines#loc-laboratory-protocols. Additionally, PLOS ONE offers an option for publishing peer-reviewed Lab Protocol articles, which describe protocols hosted on protocols.io. Read more information on sharing protocols at . Additionally, PLOS ONE offers an option for publishing peer-reviewed Lab Protocol articles, which describe protocols hosted on protocols.io. Read more information on sharing protocols at https://plos.org/protocols?utm_medium=editorial-email&utm_source=authorletters&utm_campaign=protocols..

We look forward to receiving your revised manuscript.

Kind regards,

João Miguel Dias, Ph.D.

Academic Editor

PLOS One

Journal Requirements:

When submitting your revision, we need you to address these additional requirements.

1. Please ensure that your manuscript meets PLOS ONE's style requirements, including those for file naming. The PLOS ONE style templates can be found at

https://journals.plos.org/plosone/s/file?id=wjVg/PLOSOne_formatting_sample_main_body.pdf and

https://journals.plos.org/plosone/s/file?id=ba62/PLOSOne_formatting_sample_title_authors_affiliations.pdf

2. Please note that PLOS One has specific guidelines on code sharing for submissions in which author-generated code underpins the findings in the manuscript. In these cases, we expect all author-generated code to be made available without restrictions upon publication of the work. Please review our guidelines at https://journals.plos.org/plosone/s/materials-and-software-sharing#loc-sharing-code and ensure that your code is shared in a way that follows best practice and facilitates reproducibility and reuse.

3. We note that the grant information you provided in the ‘Funding Information’ and ‘Financial Disclosure’ sections do not match.

When you resubmit, please ensure that you provide the correct grant numbers for the awards you received for your study in the ‘Funding Information’ section.

4. Thank you for stating the following financial disclosure:

This research was supported by the National Social Science Fund of China （23CXW034） ;& Fundamental Research Funds for the Central Universities: Climate Migration Types and Risk Management in Coastal Areas. (grant number B230205032); &Postgraduate Research & Practice Innovation Program of Jiangsu Province: Climate Migration Types and Risk Management in Coastal Areas. (grant number 422003151); and The Key Research Project of the National Foundation of Social Science of China: Community Governance and Post-relocation Support in Cross District Resettlement [grant number 21&ZD183].

Please state what role the funders took in the study. If the funders had no role, please state: "The funders had no role in study design, data collection and analysis, decision to publish, or preparation of the manuscript."

If this statement is not correct you must amend it as needed.

Please include this amended Role of Funder statement in your cover letter; we will change the online submission form on your behalf.

5. We note that your Data Availability Statement is currently as follows: All relevant data are within the manuscript and its Supporting Information files. Data Registration DOI: https://doi.org/10.17605/OSF.IO/V6DRJ.

Please confirm at this time whether or not your submission contains all raw data required to replicate the results of your study. Authors must share the “minimal data set” for their submission. PLOS defines the minimal data set to consist of the data required to replicate all study findings reported in the article, as well as related metadata and methods (https://journals.plos.org/plosone/s/data-availability#loc-minimal-data-set-definition).

For example, authors should submit the following data:

- The values behind the means, standard deviations and other measures reported;

- The values used to build graphs;

- The points extracted from images for analysis.

Authors do not need to submit their entire data set if only a portion of the data was used in the reported study.

If your submission does not contain these data, please either upload them as Supporting Information files or deposit them to a stable, public repository and provide us with the relevant URLs, DOIs, or accession numbers. For a list of recommended repositories, please see https://journals.plos.org/plosone/s/recommended-repositories.

If there are ethical or legal restrictions on sharing a de-identified data set, please explain them in detail (e.g., data contain potentially sensitive information, data are owned by a third-party organization, etc.) and who has imposed them (e.g., an ethics committee). Please also provide contact information for a data access committee, ethics committee, or other institutional body to which data requests may be sent. If data are owned by a third party, please indicate how others may request data access.

6. We note that Figures 2, 3, 4, 5, 6, 7, and 8 in your submission contain map images which may be copyrighted. All PLOS content is published under the Creative Commons Attribution License (CC BY 4.0), which means that the manuscript, images, and Supporting Information files will be freely available online, and any third party is permitted to access, download, copy, distribute, and use these materials in any way, even commercially, with proper attribution. For these reasons, we cannot publish previously copyrighted maps or satellite images created using proprietary data, such as Google software (Google Maps, Street View, and Earth). For more information, see our copyright guidelines: http://journals.plos.org/plosone/s/licenses-and-copyright.

We require you to either (a) present written permission from the copyright holder to publish these figures specifically under the CC BY 4.0 license, or (b) remove the figures from your submission:

a. You may seek permission from the original copyright holder of Figures 2, 3, 4, 5, 6, 7, and 8 to publish the content specifically under the CC BY 4.0 license.

We recommend that you contact the original copyright holder with the Content Permission Form (http://journals.plos.org/plosone/s/file?id=7c09/content-permission-form.pdf) and the following text:

“I request permission for the open-access journal PLOS ONE to publish XXX under the Creative Commons Attribution License (CCAL) CC BY 4.0 (http://creativecommons.org/licenses/by/4.0/). Please be aware that this license allows unrestricted use and distribution, even commercially, by third parties. Please reply and provide explicit written permission to publish XXX under a CC BY license and complete the attached form.”

Please upload the completed Content Permission Form or other proof of granted permissions as an ""Other"" file with your submission.

In the figure caption of the copyrighted figure, please include the following text: “Reprinted from [ref] under a CC BY license, with permission from [name of publisher], original copyright [original copyright year].”

b. If you are unable to obtain permission from the original copyright holder to publish these figures under the CC BY 4.0 license or if the copyright holder’s requirements are incompatible with the CC BY 4.0 license, please either i) remove the figure or ii) supply a replacement figure that complies with the CC BY 4.0 license. Please check copyright information on all replacement figures and update the figure caption with source information. If applicable, please specify in the figure caption text when a figure is similar but not identical to the original image and is therefore for illustrative purposes only.

The following resources for replacing copyrighted map figures may be helpful:

USGS National Map Viewer (public domain): http://viewer.nationalmap.gov/viewer/

The Gateway to Astronaut Photography of Earth (public domain): http://eol.jsc.nasa.gov/sseop/clickmap/

Maps at the CIA (public domain): https://www.cia.gov/library/publications/the-world-factbook/index.html and https://www.cia.gov/library/publications/cia-maps-publications/index.html

NASA Earth Observatory (public domain): http://earthobservatory.nasa.gov/

Landsat: http://landsat.visibleearth.nasa.gov/

USGS EROS (Earth Resources Observatory and Science (EROS) Center) (public domain): http://eros.usgs.gov/#

Natural Earth (public domain): http://www.naturalearthdata.com/

7. Please include a copy of Table 1 which you refer to in your text on page 8.

8. We note you have included a table to which you do not refer in the text of your manuscript. Please ensure that you refer to Table 2 in your text; if accepted, production will need this reference to link the reader to the Table.

9. If the reviewer comments include a recommendation to cite specific previously published works, please review and evaluate these publications to determine whether they are relevant and should be cited. There is no requirement to cite these works unless the editor has indicated otherwise.

[Note: HTML markup is below. Please do not edit.]

Reviewers' comments:

Reviewer's Responses to Questions

**Comments to the Author**

1. Is the manuscript technically sound, and do the data support the conclusions?

The manuscript must describe a technically sound piece of scientific research with data that supports the conclusions. Experiments must have been conducted rigorously, with appropriate controls, replication, and sample sizes. The conclusions must be drawn appropriately based on the data presented.

Reviewer #1: Partly

Reviewer #2: Partly

**********

2. Has the statistical analysis been performed appropriately and rigorously?

Reviewer #1: Yes

Reviewer #2: N/A

**********

3. Have the authors made all data underlying the findings in their manuscript fully available?

The PLOS Data policy requires authors to make all data underlying the findings described in their manuscript fully available without restriction, with rare exception (please refer to the Data Availability Statement in the manuscript PDF file). The data should be provided as part of the manuscript or its supporting information, or deposited to a public repository. For example, in addition to summary statistics, the data points behind means, medians and variance measures should be available. If there are restrictions on publicly sharing data—e.g. participant privacy or use of data from a third party—those must be specified.requires authors to make all data underlying the findings described in their manuscript fully available without restriction, with rare exception (please refer to the Data Availability Statement in the manuscript PDF file). The data should be provided as part of the manuscript or its supporting information, or deposited to a public repository. For example, in addition to summary statistics, the data points behind means, medians and variance measures should be available. If there are restrictions on publicly sharing data—e.g. participant privacy or use of data from a third party—those must be specified.

Reviewer #1: No

Reviewer #2: Yes

**********

4. Is the manuscript presented in an intelligible fashion and written in standard English?

PLOS ONE does not copyedit accepted manuscripts, so the language in submitted articles must be clear, correct, and unambiguous. Any typographical or grammatical errors should be corrected at revision, so please note any specific errors here.

Reviewer #1: Yes

Reviewer #2: Yes

**********

5. Review Comments to the Author

Please use the space provided to explain your answers to the questions above. You may also include additional comments for the author, including concerns about dual publication, research ethics, or publication ethics. (Please upload your review as an attachment if it exceeds 20,000 characters)

Reviewer #1: Rising Tides: Unveiling the Spatial and Temporal Evolution of Sea Level Rise Under Climate Change by Liang et al., submitted to PLOS One.

The manuscript presents a methodology to study the spatiotemporal evolution of seal level rise (SLR) under climate change using three different spatial scales: global, regional (China) and local (Shanghai), using IPCC AR6 dataset and NASA sea level projections.

In general, the manuscript is well written, and it is easy to understand for an international reader. Th structure of the paper is good, and the figures have good visual quality. The references used in this study represents high standard international studies and supports well the text. Despite the good quality of the grammar, the text should be edited to remove (or add spaces) between the text and sone numbers of the figures (line 219 as example).

The paper follows a logical sequence and until the presentation of the methods it is really easy to follow and understand. However, the presentation of the results is poor, and the authors choose to present the results in a very qualitative form.

In the following I will refer some positive and less positive aspects of the study, and I will try to propose some aspects to improve the study (not the study itself but its presentation).

The introduction presents what it’s known about SLR values and possible impacts and the drivers of sea level rise at different spatial scales, linking the process with thermal expansion and the melting of ice caps, following a brief description of SLR. Then, a description of what is spatiotemporal evolution and why its study is important.

The authors refer that there are few studies with high resolution results at small scale. However, in the literature there are many examples of the impact of SLR in coastal systems (in variables like salinity intrusion, inundation areas, biogeochemistry, etc), like coastal seas and estuaries throughout the world and, in my opinion, you can rewrite the introduction to present those studies.

The main objectives are well presented and described. The focus is the local scale, and it is not clear why the authors choose to do the analysis in global and regional scales and another aspect that is not clear is the link of the global and regional scales to the local scale analysis. You should improve this presentation.

The study area section presents a brief description of the local area of Shanghai. The description of the study region is made poorly. Loca elevation and maximum precipitation are described but no information regarding tidal amplitude is provided. Moreover, rivers are referred to be important, but no information about freshwater inflow values and its importance to local trends is given. More details should be included to improve this section (Study Area), which will be useful to international readers.

In section 2.2. the data sources are presented. However, in the text, only the names of the data sets are given. More information about the data sources is needed. Are the data daily, monthly, hourly? How about the spatial resolution of the data? You should refer this and then you can give the references for the data.

The authors used a method that uses the Theil-Sen Median estimator and claims that this methos is robust against outliers and noisy data. This is a fact, the authors are right, but the question here is: Why use it with SLR data? These data present outliers and a noisy temporal evolution? Despite this question, the methods are very well described.

In the results section, the authors tell us that the IPCC AR6 emission scenario are used. You should clearly present the emission scenarios that were used in this study. You should introduce these details in section 2.2.

The description of the results can be improved.in lines 219-221, the authors refer that the Seen’s slope and the MK test categorizes the trends into nine distinct classes. I believe that, but this result is not visible in Figure 3. You should show the classes explicitly, maybe with zooms for more localizes locations.

In the following I will include some comments about the presentation of the results. I think that this section should be rewritten because:

1) In Figure 3, 9 classes are displayed, but most of the classes (different from “Highly Significant Increase”) are not visible. To improve the display of the results, maybe some zooms should be generated near the coastal areas. This comment is valid to every figure showing different classification classes at global, regional or local scale.

2) The results are discussed in a rather qualitative manner. Moreover, the presentation of the results from the analysis of the data relates to phenomenon’s driving SLR (e.g. glacial melting or thermal expansion of the ocean). In these parts of the text you should introduce some references. Between lines 237 and 249, there is a whole paragraph where supporting references are needed.

3) Between lines 256 and 267, the analysis for the coast of China is presented. The comment 2) is valid for this figure. At the bottom of the page, a connection between the results and remote forcing effects is presented. However, the connection between these remote forcing and regional scale results should be supported by references to other studies where the same (or similar) class classification was described.

4) The same is applicable to the local scale of the area of Shanghai.

5) The authors uses in the manuscript two different reference types: the numeric and the classical last name of the author plus the yea. Please present the references in an uniform manner.

6) Figure 6 depicts the results for 2050, 2070 and 2100. To a clearly presentation, the authors should consider depicting differences from 2070 and 2100 to 2050 or to another reference period. This should improve the presentation of the results.

In summary, all the presentation of the results should be rewritten to achieve a better description of the results and the connection between this conclusions and other studies made for another areas of the globe.

Reviewer #2: 1. Critique of Figure 1 (Mechanism Diagram)

The current Figure 1, titled "Mechanism Diagram of SLR," is presented as a conceptual cartoon that illustrates greenhouse gas emissions, global warming, and ocean responses in a highly simplified manner. While it captures the general flow of the problem, it lacks the scientific depth and technical data expected in a research article.

Proposed Technical Diagram: To improve the manuscript's professionalism, Figure 1 should be replaced with a systems-based physical schematic. A more technical diagram should include:

• External Forcing: Explicitly labeling radiative forcing and specific Greenhouse Gases (GHGs).

• Thermodynamic Components: Differentiating between steric SLR (thermal expansion and halosteric effects) and mass-driven SLR (ice sheet discharge and glacier melt).

• Regional Modulators: Adding nodes for Glacial Isostatic Adjustment (GIA), ocean dynamic processes like the AMOC slowdown, and localized land subsidence.

• Interactions: Using technical flowlines to show how polar amplification affects seawater density and volume.

2. Restructuring of the Methodology (Section 2.3)

The presentation of the Theil-Sen Median Estimator and the Mann-Kendall Test in Section 2.3 currently resembles a technical report or a manual rather than an academic paper.

Required Improvements:

• Narrative Integration: Phrases such as "Step 1: Compute the MK Statistic S" or "Step 2: Perform Trend Test" should be converted into a continuous narrative. For example: "The statistical significance of the detected trends was evaluated using the standardized Z-statistic, derived from the variance of S..."

• Equation Formatting: Equations (1-1) through (1-7) should be better integrated into the text syntax. Every variable (e.g., n, sgn, wij) must be formally defined immediately following the equation in which it first appears.

• Unification of Variables: Ensure consistent typography for variables throughout the section to avoid the appearance of a fragmented report.

3. Additional Technical Comments

• Abstract Terminology: The abstract uses highly abstract terms such as "epistemic hybridization" and "anticipatory governance tools". While innovative, these terms should be more clearly linked to the specific quantitative results of the tri-scale framework.

• Scale Redundancy: There is significant repetition of descriptive text between the global scale (3.1.1) and China scale (3.1.2) results. Specifically, the paragraphs explaining AMOC slowdown and halosteric effects are nearly identical in both sections. Each scale analysis should focus on its unique regional drivers rather than restating global mechanisms.

• Subsidence Attribution: The study highlights land subsidence as a major driver for Shanghai's relative SLR. The manuscript would benefit from clarifying if the NASA projection models used already account for anthropogenic subsidence (e.g., groundwater extraction) or if this was a separate parameter integrated by the authors.

• Visualization Consistency: The maps in Figures 3, 4, and 5 use a 9-class trend classification. Providing a summary table with the specific median slope values (β) for key regions would provide the quantitative evidence necessary to support the qualitative "Hotspot" descriptions

**********

6. PLOS authors have the option to publish the peer review history of their article (what does this mean?). If published, this will include your full peer review and any attached files.). If published, this will include your full peer review and any attached files.

If you choose “no”, your identity will remain anonymous but your review may still be made public.

**Do you want your identity to be public for this peer review?**  For information about this choice, including consent withdrawal, please see our For information about this choice, including consent withdrawal, please see our Privacy Policy..

Reviewer #1: No

Reviewer #2: No

**********

[NOTE: If reviewer comments were submitted as an attachment file, they will be attached to this email and accessible via the submission site. Please log into your account, locate the manuscript record, and check for the action link "View Attachments". If this link does not appear, there are no attachment files.]

To ensure your figures meet our technical requirements, please review our figure guidelines: https://journals.plos.org/plosone/s/figures

You may also use PLOS’s free figure tool, NAAS, to help you prepare publication quality figures: https://journals.plos.org/plosone/s/figures#loc-tools-for-figure-preparation.

NAAS will assess whether your figures meet our technical requirements by comparing each figure against our figure specifications.

# Author comment: Author response to Decision Letter 1

**DOI:** 10.1371/journal.pone.0347855.r002

17 Mar 2026

ok

Attachment Submitted filename: Response to Reviewers.pdf

# Peer review: Decision Letter 1

**DOI:** 10.1371/journal.pone.0347855.r003

**Authors:** João Miguel Dias

8 Apr 2026

Rising Tides: Unveiling the Spatial and Temporal Evolution of Sea Level Rise Under Climate Change

PONE-D-25-55574R1

Dear Dr. Liang,

We’re pleased to inform you that your manuscript has been judged scientifically suitable for publication and will be formally accepted for publication once it meets all outstanding technical requirements.

Within one week, you’ll receive an e-mail detailing the required amendments. When these have been addressed, you’ll receive a formal acceptance letter and your manuscript will be scheduled for publication.

An invoice will be generated when your article is formally accepted. Please note, if your institution has a publishing partnership with PLOS and your article meets the relevant criteria, all or part of your publication costs will be covered. Please make sure your user information is up-to-date by logging into Editorial Manager at Editorial Manager® and clicking the ‘Update My Information' link at the top of the page. For questions related to billing, please contact  and clicking the ‘Update My Information' link at the top of the page. For questions related to billing, please contact billing support..

If your institution or institutions have a press office, please notify them about your upcoming paper to help maximize its impact. If they’ll be preparing press materials, please inform our press team as soon as possible -- no later than 48 hours after receiving the formal acceptance. Your manuscript will remain under strict press embargo until 2 pm Eastern Time on the date of publication. For more information, please contact onepress@plos.org.

Kind regards,

João Miguel Dias, Ph.D.

Academic Editor

PLOS One

Additional Editor Comments (optional):

Reviewers' comments:

Reviewer's Responses to Questions

**Comments to the Author**

1. If the authors have adequately addressed your comments raised in a previous round of review and you feel that this manuscript is now acceptable for publication, you may indicate that here to bypass the “Comments to the Author” section, enter your conflict of interest statement in the “Confidential to Editor” section, and submit your "Accept" recommendation.

Reviewer #1: All comments have been addressed

**********

2. Is the manuscript technically sound, and do the data support the conclusions?

The manuscript must describe a technically sound piece of scientific research with data that supports the conclusions. Experiments must have been conducted rigorously, with appropriate controls, replication, and sample sizes. The conclusions must be drawn appropriately based on the data presented.

Reviewer #1: Yes

**********

3. Has the statistical analysis been performed appropriately and rigorously?

Reviewer #1: Yes

**********

4. Have the authors made all data underlying the findings in their manuscript fully available?

The PLOS Data policy requires authors to make all data underlying the findings described in their manuscript fully available without restriction, with rare exception (please refer to the Data Availability Statement in the manuscript PDF file). The data should be provided as part of the manuscript or its supporting information, or deposited to a public repository. For example, in addition to summary statistics, the data points behind means, medians and variance measures should be available. If there are restrictions on publicly sharing data—e.g. participant privacy or use of data from a third party—those must be specified.requires authors to make all data underlying the findings described in their manuscript fully available without restriction, with rare exception (please refer to the Data Availability Statement in the manuscript PDF file). The data should be provided as part of the manuscript or its supporting information, or deposited to a public repository. For example, in addition to summary statistics, the data points behind means, medians and variance measures should be available. If there are restrictions on publicly sharing data—e.g. participant privacy or use of data from a third party—those must be specified.

Reviewer #1: Yes

**********

5. Is the manuscript presented in an intelligible fashion and written in standard English?

PLOS ONE does not copyedit accepted manuscripts, so the language in submitted articles must be clear, correct, and unambiguous. Any typographical or grammatical errors should be corrected at revision, so please note any specific errors here.

Reviewer #1: Yes

**********

6. Review Comments to the Author

Please use the space provided to explain your answers to the questions above. You may also include additional comments for the author, including concerns about dual publication, research ethics, or publication ethics. (Please upload your review as an attachment if it exceeds 20,000 characters)

Reviewer #1: Rising Tides: Unveiling the Spatial and Temporal Evolution of Sea Level Rise Under Climate Change by Liang et al., re-submitted to PLOS One.

The manuscript presents a methodology to study the spatiotemporal evolution of seal level rise (SLR) under climate change using three different spatial scales: global, regional (China) and local (Shanghai), using IPCC AR6 dataset and NASA sea level projections.

In general, the manuscript is well written, and it is easy to understand for an international reader. It’s structure is good, and the figures have good visual quality. The references used in this study represents high standard international studies and supports well the text.

The paper follows a logical sequence and until the presentation of the methods it is really easy to follow and understand. However, the presentation of the results is poor, and the authors choose to present the results in a very qualitative form.

Generally, all comments and suggestions have been addressed, with particular attention given to improving the clarity, structure, and completeness of the presentation of the methods and results. The introduction has been revised to better incorporate relevant literature on sea level rise impacts in coastal and estuarine systems, and to strengthen the link between global, regional, and local scales. The study area section has been expanded to include additional key information—such as tidal dynamics and freshwater inputs—ensuring a more comprehensive description accessible to an international audience. Furthermore, the data sources are now described in greater detail, including their temporal and spatial resolution, and the emission scenarios adopted from IPCC AR6 have been clearly specified.

In addition, the authors have carefully revised the methodology and results sections to improve transparency and interpretability. The rationale for using the Theil–Sen estimator has been clarified in the context of sea level data characteristics, and the presentation of results has been substantially enhanced. Additional references have been incorporated to support the interpretation of observed trends and their driving mechanisms. The discussion now provides a clearer connection between results at different spatial scales and previously published studies. Finally, the manuscript has been edited to ensure consistency in the citation style and to improve the overall coherence and readability of the results section.

**********

7. PLOS authors have the option to publish the peer review history of their article (what does this mean?). If published, this will include your full peer review and any attached files.). If published, this will include your full peer review and any attached files.

If you choose “no”, your identity will remain anonymous but your review may still be made public.

**Do you want your identity to be public for this peer review?**  For information about this choice, including consent withdrawal, please see our For information about this choice, including consent withdrawal, please see our Privacy Policy..

Reviewer #1: No

**********

# Editor report: Acceptance letter

**DOI:** 10.1371/journal.pone.0347855.r004

**Authors:** João Miguel Dias

PONE-D-25-55574R1

PLOS One

Dear Dr. Liang,

I'm pleased to inform you that your manuscript has been deemed suitable for publication in PLOS One. Congratulations! Your manuscript is now being handed over to our production team.

At this stage, our production department will prepare your paper for publication. This includes ensuring the following:

* All references, tables, and figures are properly cited

* All relevant supporting information is included in the manuscript submission,

* There are no issues that prevent the paper from being properly typeset

You will receive further instructions from the production team, including instructions on how to review your proof when it is ready. Please keep in mind that we are working through a large volume of accepted articles, so please give us a few days to review your paper and let you know the next and final steps.

Lastly, if your institution or institutions have a press office, please let them know about your upcoming paper now to help maximize its impact. If they'll be preparing press materials, please inform our press team within the next 48 hours. Your manuscript will remain under strict press embargo until 2 pm Eastern Time on the date of publication. For more information, please contact onepress@plos.org.

You will receive an invoice from PLOS for your publication fee after your manuscript has reached the completed accept phase. If you receive an email requesting payment before acceptance or for any other service, this may be a phishing scheme. Learn how to identify phishing emails and protect your accounts at https://explore.plos.org/phishing.

If we can help with anything else, please email us at customercare@plos.org.

Thank you for submitting your work to PLOS ONE and supporting open access.

Kind regards,

PLOS ONE Editorial Office Staff

on behalf of

Prof. João Miguel Dias

Academic Editor

PLOS One
